# A platform for post-translational spatiotemporal control of cellular proteins

**DOI:** 10.1093/synbio/ysab002

**Published:** 2021-02-02

**Authors:** Brianna Jayanthi, Bhagyashree Bachhav, Zengyi Wan, Santiago Martinez Legaspi, Laura Segatori

**Affiliations:** 1 Systems, Synthetic and Physical Biology Graduate Program, Rice University, Houston, TX, USA; 2 Department of Chemical and Biomolecular Engineering, Rice University, Houston, TX, USA; 3 Department of Bioengineering, Rice University, Houston, TX, USA; 4 Department of Biosciences, Rice University, Houston, TX, USA

**Keywords:** degradation, localization, mammalian genetic circuits, nanobody, orthogonal protein regulation

## Abstract

Mammalian cells process information through coordinated spatiotemporal regulation of proteins. Engineering cellular networks thus relies on efficient tools for regulating protein levels in specific subcellular compartments. To address the need to manipulate the extent and dynamics of protein localization, we developed a platform technology for the target-specific control of protein destination. This platform is based on bifunctional molecules comprising a target-specific nanobody and universal sequences determining target subcellular localization or degradation rate. We demonstrate that nanobody-mediated localization depends on the expression level of the target and the nanobody, and the extent of target subcellular localization can be regulated by combining multiple target-specific nanobodies with distinct localization or degradation sequences. We also show that this platform for nanobody-mediated target localization and degradation can be regulated transcriptionally and integrated within orthogonal genetic circuits to achieve the desired temporal control over spatial regulation of target proteins. The platform reported in this study provides an innovative tool to control protein subcellular localization, which will be useful to investigate protein function and regulate large synthetic gene circuits.

## 1. Introduction

Cellular information processing is primarily managed by regulating protein concentration in space and time (1, 2). Temporal regulation of protein concentration is mostly achieved through activation of transcription factors and protein degradation, which are controlled through a myriad of well-studied mechanisms (3–12). Proteins are typically synthesized in the cytosol or on ER-bound ribosomes and trafficked to their final destination based on signal sequences contained in the protein’s primary sequence (13). Protein function depends on subcellular location and is drastically affected by environmental conditions, such as pH and redox state (14–17). Protein localization also dictates the availability of interacting proteins (18–21) and substrates (22–24), further diversifying protein functionality. Moreover, many cellular responses are regulated by mechanisms controlling changes in localized protein concentration, including translocation of transcription factors from the cytosol to the nucleus (25), release of mitochondrial proteins into the cytosol during apoptosis (26), endocytic recycling of plasma membrane proteins (27) and scaffold recruitment and release of signaling pathway components (28). Spatial organization of cellular components is also of vital importance in cellular manufacturing of proteins and metabolites (29), as it provides a mechanism for accelerating reactions (30, 31) and reducing interference between pathways with shared components (32). Furthermore, aberrant localization of proteins has been associated with disease states including neurodegeneration (33–35), metabolic impairment (36, 37) and cancer (38, 39). Controlling protein localization would thus open the way to a number of applications ranging from fundamental studies of protein function to therapeutic protein delivery. Previous attempts to target proteins to specific compartments have facilitated characterization studies (40), manipulation of protein activity (41, 42) and control of metabolic pathways (43, 44). Perturbing cellular protein levels and monitoring the associated phenotypic changes are widely recognized as a powerful approach to investigate biological pathways (45–47). Similarly, characterization of the physiological role of protein localization in the complex milieu of mammalian cells depends on tools to precisely perturb the location of cellular proteins and monitor the associated phenotypic changes (48–50).

Control of protein localization can be achieved through fusion of a protein of interest to well-characterized sequences known to control protein trafficking (13, 51). This approach, however, requires genetic manipulations of the target protein, which may potentially affect the protein’s native function (52, 53). In addition, the efficiency of protein localization is largely target specific (54, 55). Control over the location of a target protein can also be achieved through engineered inducible interactions between a protein residing at the location of interest and the target protein. This approach to regulate protein localization requires extensive genetic manipulation and is generally implemented through fusion of a dimerizing domain to a protein at the desired location and to the target protein. Small molecule (40, 41, 56–58) or light (59–61) inducible dimerization has been widely used to control target localization. These systems, however, are limited by the requirement for a binding partner with the correct subcellular address and that is amenable to protein engineering. Moreover, regulating localization of proteins based on fusion to a signal sequence or dimerization domain is a cumbersome endeavor due to the need to modify each target individually. Furthermore, the availability of a restricted number of inducible dimerization domains (56) limits the development of strategies for directing single targets to multiple compartments or for multiplexing the control of targets. An alternative approach to control the location of cellular proteins relies on the use of binding proteins (DARPins, antibody fragments and nanobodies) functionalized with localization signals (50, 62–64). While target-specific recognition molecules engineered to control target localization provide powerful tools to investigate the role of protein subcellular localization without modifying the target protein, current approaches based on recognition molecule-mediated localization do not allow for user-defined control over the extent and dynamics of target localization.

In this study, we addressed the need for a platform technology for regulating the localization of cellular proteins that can be potentially adapted to any cellular target protein, provides dynamic control of the protein levels at the desired subcellular location and allows modulating subcellular localization of a single target to multiple compartments. In this regard, we constructed a nanobody-based platform for the tunable control of target protein localization. Specifically, we built a toolbox of GFP-specific nanobodies functionalized to direct a target to a series of subcellular compartments (NanoLoc). We demonstrated that nanobody-mediated localization of a target protein depends on the expression level of the nanobody and of the target protein. We also provided proof-of-principle demonstration of a universal NanoLoc targeting a peptide tag (BC2) (65) for controlling the function of a BC2-tagged transcription factor via targeted localization. We developed a method to modulate the extent of target subcellular localization based on combining the use of target-specific nanobodies mediating localization (VHH_Loc_) and degradation (VHH_Deg_) (66). Finally, we interfaced this toolkit for nanobody-mediated control with different strategies for transcriptional regulation of the nanobody to achieve spatial and temporal control of the target protein.

## 2. Materials and methods

### 2.1 Plasmids and cloning

Localization signal sequences are listed in Supplementary Table S1. The plasmids and primers used in this study are listed in Supplementary Tables S2 and S3, respectively. Plasmids were transformed and maintained in Stbl3 *E. coli* competent cells (Thermo Fisher Scientific). PCR amplifications of DNA fragments were performed using Kappa HiFi DNA polymerase (Kapa Biosystems) according to the manufacturer’s protocol. Plasmid sequences are available in the Supplementary data.

The plasmid pSV40-GFP was generated by replacing the CMV promoter of pLenti CMV GFP Blast (Addgene plasmid # 17445) with the SV40 promoter using restriction enzymes ClaI and BamHI. The neomycin-resistance gene was PCR amplified from pcDNA3.1+ (Invitrogen) with primers designed to add BamHI and BlpI enzyme recognition sites 5′ and 3′, respectively. The resulting fragment was ligated into the pLenti SV40 GFP vector, replacing the blasticidin-resistance gene, after digestion with BamHI and BlpI restriction enzymes.

The GFP-specific VHH encoding sequence with a 5′ fused HA tag sequence was amplified from pvhh (66). The pCMV-VHH_MOM_, pCMV-VHH_ERM_, pCMV-VHH_PEX_ and pCMV-VHH_NLS_ were constructed using overlap extension PCR to fuse the localization signal sequence 3′ of the HA-VHH encoding sequence. The PCR amplified fragments encoding HA-VHH_Loc_ variants were ligated into the pvhh vector (66) after digestion with KpnI and NotI restriction enzymes. To generate pCMV-VHH_PM_, the sequence encoding the plasma membrane localization signal was fused to the 5′ end of the *vhh* and the HA tag encoding sequence was fused to the 3′ end of the *vhh* using overlap extension PCR. The resulting sequence was ligated into the pvhh vector after digestion with AflII and NotI restriction enzymes.

The sequence encoding the BC2 tag (65) was obtained from Integrated DNA technologies and PCR amplified with primers designed to add NotI and XhoI enzyme recognition sites 5′ and 3′, respectively. The resulting fragment was ligated into the pCMV-TetR vector (Invitrogen) after digestion with NotI and XhoI restriction enzymes, generating the plasmid pCMV-TetR-BC2T. The *irfp* sequence was PCR amplified from piRFP (Addgene plasmid # 31857) with primers designed to add NheI and NotI enzyme recognition sites 5′ and 3′, respectively. The pCMV-iRFP-BC2T plasmid was generated from pCMV-TetR-BC2T by replacing *tetR* with *irfp* using NheI and NotI restriction enzymes. The *tTA* sequence was PCR amplified from ptTA (67) with primers designed to add NheI and NotI enzyme recognition sites 5′ and 3′, respectively. The pCMV-tTA-BC2T plasmid was generated from pCMV-iRFP-BC2T by replacing *irfp* with *tTA* using NheI and NotI restriction enzymes. The sequence encoding BC2 tag-specific VHH (65) with a 5′ HA tag fusion was obtained from Integrated DNA technologies. The resulting sequence (VHH^BC2T^) was PCR amplified with primers designed to add KpnI and BlpI restriction sites at the 5′ and 3′, respectively. The pCMV-${\mathrm{VHH}}_{\mathrm{NLS}}^{\mathrm{BC}2T}$ was constructed by excising the VHH_NLS_ sequence from the pCMV-VHH_NLS_ and replacing it with VHH^BC2T^, using restriction enzymes KpnI and BlpI.

The pCMV-TetR-IRES-EKRAB plasmid was constructed by ligating *TetR* (Invitrogen), an IRES (Addgene plasmid # 21547) and *EKRAB* (68) into the pLenti CMV eGFP Zeo vector (Addgene plasmid # 17449) after restriction enzyme digestion of *TetR* with SpeI and SbfI, IRES with SbfI and MluI and *EKRAB* with MluI and SalI.

The pCMV/TO-iRFP plasmid was generated from pcDNA4/TO (Invitrogen) by replacing *gfp* with *irfp* (piRFP, Addgene plasmid # 31857) using KpnI and NotI restriction enzymes. The CMV promoter containing four downstream repeats of the ETR operator site (68) (CMV/ETR) was generated by assembly PCR. The CMV/ETR promoter sequence was ligated into the pCMV/TO-iRFP vector, replacing the CMV/TO promoter, after digestion with MluI and KpnI restriction enzymes. The pCMV/TO-VHH_MOM_ plasmid was constructed by excising the HA-VHH_MOM_ sequence from pCMV-VHH_MOM_ and ligating it into pCMV/TO-iRFP, replacing *irfp*, using restriction enzymes KpnI and NotI. The pCMV/TO-VHH_NLS_ plasmid was constructed in the same manner by excising VHH_NLS_ from pCMV-VHH_NLS_. The pCMV/ETR.VHH_ODC(wt)_ plasmid was constructed by excising VHH_ODC(wt)_ from pvhh-ODC (66) and ligating it into the pCMV/ETR-iRFP backbone, replacing *irfp*, using restriction enzymes KpnI and NotI. The pCMV/ETR-VHH_MOM_ plasmid was constructed in the same manner by excising VHH_MOM_ from pCMV-VHH_MOM_.

To construct pCMV/ETR-VHH_NLS_-IRES-PIPKRAB, the insert sequence encoding HA-VHH_NLS_-IRES-PIPKRAB was generated by PCR amplification of HA-VHH_NLS_ from pCMV-VHH_NLS_ with primers designed to add the NheI and AgeI enzyme recognition sites at the 5′ and 3′ ends, respectively. The IRES sequence was amplified from pCMV-TetR-IRES-EKRAB with primers designed to add the AgeI and AflII enzyme recognition sites at the 5′ and 3′ ends, respectively, and the PIPKRAB sequence (69) was amplified with primers designed to add the AflII and NotI enzyme recognition sites at the 5′ and 3′ ends, respectively. These three insert sequences were then ligated into the pCMV/ETR-iRFP backbone, replacing *irfp*, using NheI and NotI restriction enzymes.

The pCMV/PIR-VHH_MOM_-IRES-EKRAB plasmid was constructed by first replacing the ETR operator sites of pCMV/ETR-iRFP with PIR operator sites generated by assembly PCR (69) to yield pCMV/PIR-iRFP. The sequence encoding VHH_MOM_ was PCR amplified from pCMV-VHH_MOM_ with overlapping primers designed to add a FLAG tag encoding sequence and a KasI enzyme recognition site at the 5′ end and the AgeI enzyme recognition site at the 3′ end. The IRES-EKRAB sequence was PCR amplified from pCMV.TetR-IRES-EKRAB with primers designed to add the AgeI and NotI enzyme recognition sites at the 5′ and 3′ ends, respectively. The FLAG-VHH_MOM_ and IRES-EKRAB inserts were then ligated into the pCMV/PIR-iRFP backbone, replacing *irfp*, using KasI and NotI restriction enzymes.

### 2.2 Cell culture and transfections

HEK293 cells (ATCC) and HEK293T cells (ATCC) were cultured in Dulbecco’s modified eagle medium–/high glucose (DMEM; Hyclone) supplemented with 10% fetal bovine serum (FBS; GenClone) and 1% penicillin–streptomycin–glutamine (Hyclone) and maintained at 37°C and 5% CO_2_. Cells were passaged using phosphate-buffered saline (PBS; Lonza) and trypsin (TrypLE; GIBCO Invitrogen).

Transient transfections were performed by seeding cells onto 24-well plates, 12-well plates or 10-cm tissue culture dishes. After 24 h, cells were transfected using JetPrime (Polyplus transfection) according to manufacturer’s protocol. The medium was replaced with fresh medium 8 or 16 h post-transfection and cells were replated onto coverslips 48 h post-transfection at a density of 18 × 10^4^ cells/ml. Cells were fixed for analysis at 72 h post-transfection unless otherwise indicated.

### 2.3 Lentivirus production and transduction

Third-generation lentiviruses were generated by seeding HEK293T cells onto 10-cm tissue culture dishes at a density of 10 × 10^4^ cells/ml. Cells were transfected with pSV40-GFP or pCMV-TetR-IRES-EKRAB, and the packaging plasmids pMLg/PRRE (Addgene plasmid # 12251), pRSV-Rev (Addgene plasmid # 12253) and pMD2.g (Addgene plasmid # 12259) in a 2:5:2.5:3 ratio, respectively. The medium was replaced with fresh medium 8 h post-transfection and the virus-containing medium was collected after 48 h. The virus was concentrated using a Lenti-X concentrator (Clontech) according to manufacturer’s protocol.

Cell transductions were conducted by seeding HEK293 cells onto 12-well plates at a density of 10 × 10^4^ cells/ml. After 24 h, the medium was replaced with medium supplemented with virus particles and 8 μg/ml polybrene. The medium supplemented with virus was replaced with fresh medium 24 h post-transduction.

### 2.4 Generation of stable cell lines

To generate the GFP stable cell lines (HEK293/GFP#1, HEK293/GFP#2 and HEK293/GFP#3), HEK293 cells were seeded onto 12-well plates and transduced with pSV40-GFP. Cells were transferred into 10-cm tissue culture dishes 48 h post-transduction and selected for 2 weeks using 1 mg/ml geneticin (MilliporeSigma). Cells were analyzed with a Nanocellect (Wolf) to sort cells presenting different levels of GFP fluorescence. Sorted cells were seeded onto 96-well plates containing DMEM with 20% FBS at a density of 1 cell/well, expanded, and monoclonal cell populations analyzed by flow cytometry to select the three cell lines presenting different levels of GFP fluorescence used in this study.

To generate the dual-input stable cell line (HEK293/GFP2R), HEK293/GFP#1 cells were seeded onto 12-well plates and transduced with pCMV-TetR-IRES-EKRAB. Cells were transferred into 10-cm tissue culture dishes 48 h post-transduction and selected for 2 weeks using 500 µg/ml Zeocin (InvivoGen). The selected polyclonal population was diluted onto 96-well plates at a concentration of 0.5 cells/well and expanded. Monoclonal populations were then screened by transfection with pCMV/ETR-iRFP and pCMV/TO-iRFP and treated with Em and tetracycline (Tc). The iRFP fluorescence of cells treated with or without inducer was measured by flow cytometry. The cell population exhibiting the greatest range of iRFP fluorescence intensity upon treatment with both Em and Tc was selected for further experiments.

### 2.5 Flow cytometry analyses

Cells were analyzed with a FACSCanto II flow cytometer (BD Biosciences). GFP fluorescence intensity was detected using a 488-nm laser and a 530/30-nm emission filter. iRFP fluorescence intensity was detected using a 635-nm laser and a 780/60-nm emission filter. At least 10 000 cell events were recorded in each sample for analysis.

### 2.6 Immunofluorescence for confocal microscopy

Cells were seeded onto coverslips in a 24-well plate at a density of 9 × 10^4^ cells. To stain mitochondria and plasma membrane, samples were stained with MitoTracker dye (200 nM, ThermoFisher) or Membrite dye (Biotium) prior to fixing 24 h post-seeding according to the manufacturer’s protocol. Cells were then washed once with PBS, fixed with 4% paraformaldehyde (15 min) and permeabilized with 0.1% TritonX-100 (7 min). Cells were then washed three times with 0.1% Tween-20 in PBS (PBST) and blocked with 8% BSA (Calbiochem, 2930) for 1 h. Cells were washed three times with PBST and incubated in primary antibody (rabbit anti-HA SantaCruz Biotechnology sc-805, 1:250; mouse anti-calnexin, ThermoFisher MA3-027, 1:50; mouse anti-PMP70, Novus Biologicals NBP2-36770, 1:50) overnight. Cells were then washed three times with PBST and incubated with appropriate secondary antibodies (goat anti-rabbit DyLight 549 conjugated, Rockland 611-142-002, 1:500; goat anti-rabbit DyLight 405 conjugated, Rockland 611-146-002, 1:200; goat anti-mouse DyLight 549 conjugated, KPL 072-04-18-06, 1:500) for 2 h. Hoechst 3342 nuclear stain (Thermo Fisher Scientific) was added along with secondary antibodies where indicated. Coverslips were mounted onto glass slides using anti-fade solution (Invitrogen, S36936). Images were obtained with a Nikon A-1 confocal microscope (Nikon) and the Nikon NIS Element C imaging software. The acquired images were minimally processed using ImageJ software to adjust brightness and contrast where indicated (70).

### 2.7 Image analysis

Analyses of GFP fluorescence intensity in 8-bit images were conducted using ImageJ. The areas of interest (cell and compartment) were segmented by establishing a threshold based on pixel intensity in the appropriate color channel for each image. The area of interest corresponding to the cell was first segmented in the color channel showing iRFP fluorescence, and then, the area of interest corresponding to the subcellular compartment was further segmented within the cell area of interest in the color channel corresponding to the compartment stain. The fluorescence intensity was quantified by calculating the mean pixel intensity within the segmented subcellular compartment area of interest in the color channel showing GFP fluorescence. At least 15 cells per sample were analyzed. ImageJ macros are available at https://github.com/SegatoriLab/NanoLoc.

### 2.8 Statistical analysis

All data are reported as mean and error bars represent the standard error of at least three independent experiments. Statistical significance between samples was calculated using an unpaired two-tailed Student’s *t*-test.

## 3. Results

### 3.1 Design of a nanobody-based platform for spatial control of GFP

We aimed to build a platform technology for controlling protein subcellular localization that could be adapted to target potentially any cellular protein to any cellular compartment. As a proof-of-principle demonstration, we built a set of GFP-specific nanobody (VHH) (71) variants fused to different localization signals, namely the mitochondrial outer membrane anchor (VHH_MOM_) (72), the endoplasmic reticulum membrane anchor (VHH_ERM_) (73), the peroxisome targeting signal (VHH_PEX_) (74), the plasma membrane anchor (VHH_PM_) (75) and the nuclear localization signal (VHH_NLS_) (76) (Supplementary Figure S1 and Supplementary Table S1). All nanobody variants (VHH_Loc_) contained the HA tag for detection. Specifically, the mitochondrial outer membrane anchor is a 39 amino acid C-terminal transmembrane segment of the OMP25 protein that mediates protein localization to the mitochondrial outer membrane (72, 77); the endoplasmic reticulum targeting signal is a C-terminal segment of the protein tyrosine phosphate (PTP1B), which contains a single stretch transmembrane domain and undergoes post-translational attachment to the ER membrane network (73); the peroxisome targeting signal is a C-terminal dodecamer that interacts with the tetratricopeptide repeat domain of PEX5 receptor and initializes a peroxisomal translocation event via interaction with the PEX5 proteins (74); the plasma membrane anchor consists of a sequence encoding the first 15 amino acids of Gnai2 (G Protein Subunit Alpha I2) containing *N*-myristol and *S*-palmitoyl motifs that mediates protein localization to the plasma membrane (75); and the nuclear localization signal is a seven-amino-acid sequence derived from the SV40 large T antigen that promotes the nuclear transport of otherwise cytoplasmic proteins (76).

**Figure 1.**
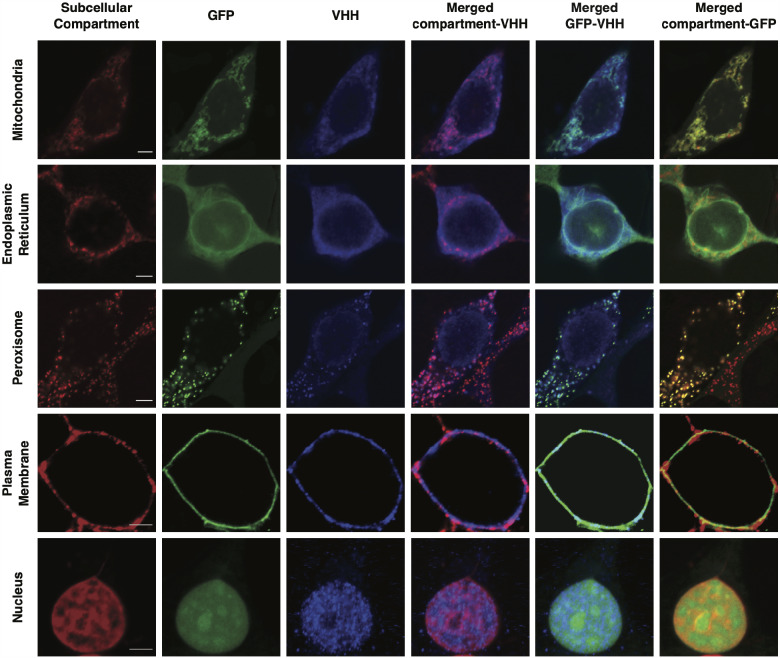
NanoLoc-mediated control of GFP subcellular localization. Representative confocal microscopy images of HEK293/GFP#1 cells transiently transfected for the expression of VHH_Loc_ variants and analyzed 72 h post-transfection. Subcellular compartment (red, column 1); GFP (green, column 2); VHH (blue, anti-HA, column 3); colocalization of subcellular compartment and VHH shown in merged images (purple, column 4); colocalization of GFP and VHH shown in merged images (cyan, column 5); colocalization of subcellular compartment and GFP shown in merged images (yellow, column 6). Scale bars: 5 µm. Brightness and contrast levels were adjusted and images of cells treated the same were subjected to the same adjustment. Pseudo-coloring was applied to the subcellular compartment stain and VHH images for the plasma membrane and the nucleus.

To verify that the resulting VHH_Loc_ variants localize cytosolic GFP to the corresponding subcellular compartments, we analyzed the colocalization of VHH_Loc_, GFP and the subcellular compartment designated by the localization tag. Specifically, HEK293 cells stably expressing GFP (HEK293/GFP#1) were transiently transfected for the expression of the VHH_Loc_ variants to evaluate GFP localization due to the VHH_Loc_ as dictated by the localization signal. Cells were analyzed by confocal microscopy for the detection of GFP, VHH (anti-HA) and the subcellular compartments, namely the mitochondria (MitoTracker stain), endoplasmic reticulum membrane (anti-CANX), peroxisome (anti-PMP70), plasma membrane (MemBrite stain) or nucleus (Hoechst stain). Imaging analyses revealed a distinct GFP and VHH distribution pattern in cells transfected for the expression of each VHH_Loc_. Specifically, the VHH_Loc_ signal was found to colocalize with the subcellular compartment marker, indicating that the localization signal controls the VHH subcellular localization (Figure 1, merged compartment-VHH, purple). GFP and VHH_Loc_ signals also colocalized, suggesting the formation of the GFP-VHH complex (Figure 1, merged GFP-VHH, cyan). Finally, colocalization of GFP and the subcellular compartment marker indicates that VHH_Loc_ controls GFP localization (Figure 1, merged compartment-GFP, yellow). Cells transfected with a control plasmid lacking the gene encoding the nanobody display a diffuse GFP signal that does not colocalize with the fluorescent signal of any of the compartment markers (Supplementary Figure S2), confirming that VHH_Loc_ controls GFP localization to the compartment designated by the localization signal.

**Figure 2.**
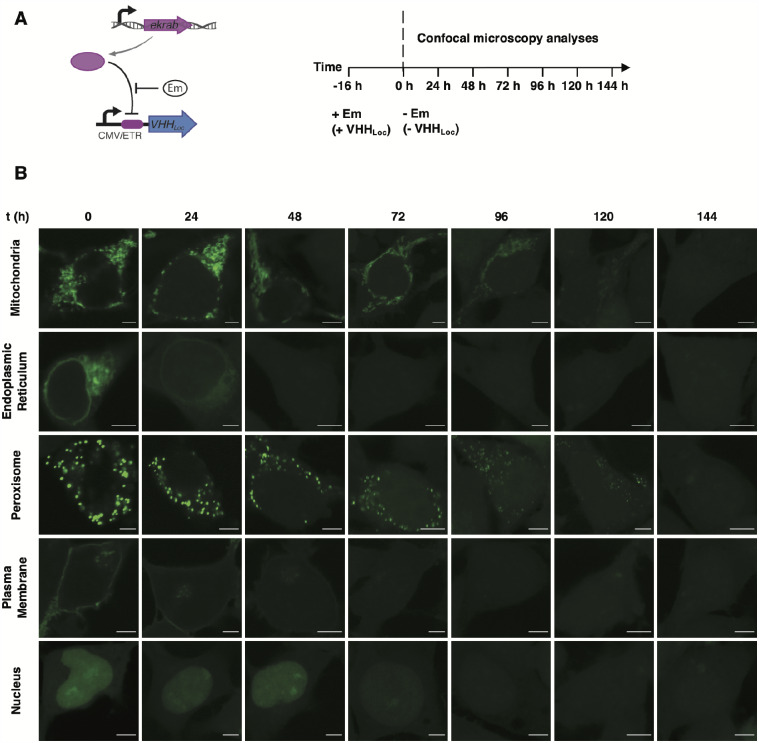
Residence time of NanoLoc-mediated GFP subcellular localization. (**A**) Schematic representation of the NanoLoc platform (left). Expression of VHH_Loc_ is induced upon the addition of Em, which displaces the Em-dependent transrepressor (EKRAB) from the Em operator (ETR). EKRAB is expressed constitutively. Cells were treated with Em for 16 h to induce expression of VHH_Loc_. Confocal microscopy analyses were initiated at the time of removal of Em (*t* = 0) from the culturing medium (right). (**B**) Representative confocal microscopy images of HEK293/GFP2R cells transiently transfected for the expression of VHH_Loc_ variants and induced with Em (500 ng/ml) 8 h post-transfection. Time-course analyses were initiated at the time of Em removal (*t* = 0 h) and conducted every 24 h for 144 h. Scale bars: 5 µm. Brightness levels were adjusted and images of cells treated the same were subjected to the same adjustment.

To test the residence time of GFP within each subcellular compartment, we monitored GFP fluorescence over time in cells expressing the VHH_Loc_ variants. HEK293/GFP#1 cells stably expressing the erythromycin (Em)-dependent transrepressor (EKRAB) (68) were transiently transfected for the expression of each VHH_Loc_ under the control of the Em-inducible operator and treated with Em to induce the expression of VHH_Loc_ (Figure 2A). Confocal microscopy analyses were initiated at the time of removal of Em from the culturing medium. Localization of GFP to the ER membrane and plasma membrane decayed rapidly with complete loss of localization by 48 and 72 h, respectively. Nuclear localization of GFP appeared stable for the first 72 h, followed by a slow decay and complete loss of localization by 120 h. Peroxisomal and mitochondrial localization presented similar temporal patterns resulting in the total loss of localization by 144 h (Figure 2B). These results are indicative of a compartment-specific localization decay rate, which may be due to a multitude of factors including different compartment turnover rates and localization mechanisms.

**Figure 3.**
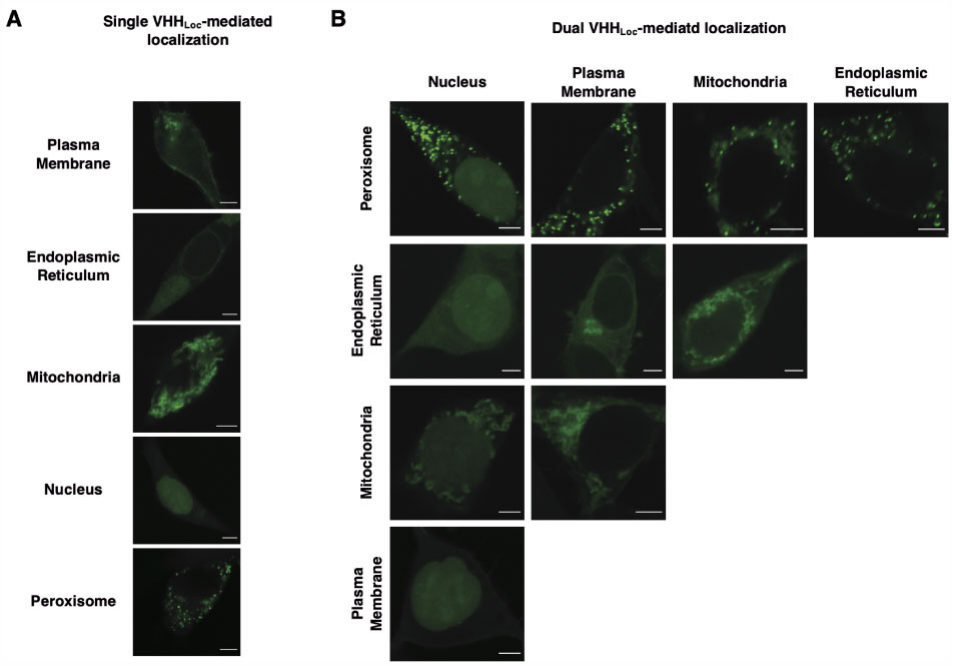
NanoLoc-mediated control of GFP subcellular localization upon expression of multiple VHH_Loc_ variants. Representative confocal microscopy images of HEK293/GFP#1 cells transiently transfected for the expression of (**A**) a single VHH_Loc_ variant and (**B**) two VHH_Loc_ variants using a 1:1 plasmid ratio. Samples were analyzed 72 h post-transfection. Scale bars: 5 µm. Brightness levels were adjusted and images of cells treated the same were subjected to the same adjustment.

To test whether GFP can be simultaneously targeted to multiple subcellular compartments, HEK293/GFP#1 cells were co-transfected for the expression of two VHH_Loc_ variants fused to different localization signals. Expression of a single VHH_Loc_ displayed the pattern of GFP fluorescence consistent with GFP localization to the designated compartment (Figure 3A). Expression of two distinct VHH_Loc_, however, resulted in a pattern of GFP fluorescence consistent with GFP localization in two distinct compartments for all combinations of VHH_Loc_ tested (Figure 3B). These results indicate that a cytosolic protein can be localized simultaneously to multiple subcellular compartments through co-expression of the appropriate VHH_Loc_ variants.

**Figure 4.**
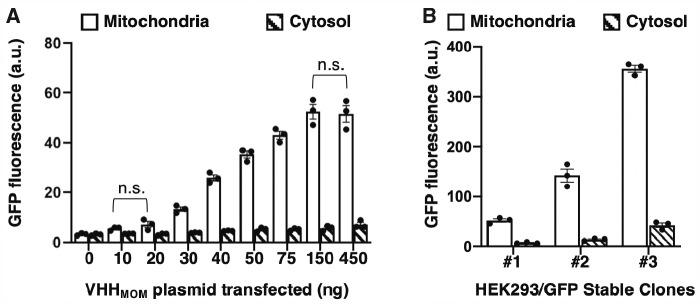
GFP mitochondrial localization as a function of VHH_MOM_ and GFP expression level. (**A**) Mitochondria-localized and cytosolic GFP fluorescence of HEK293/GFP#1 cells transiently transfected with plasmid expressing VHH_MOM_ (0–450 ng) and analyzed 72 h post-transfection by confocal microscopy. Mitochondria-localized GFP fluorescence intensity values were obtained by quantifying the GFP signal that co-localizes with the MitoTracker stain. Data are reported as mean ± s.e.m. (*n* = 3, *P *<* *0.05, Student’s *t*-test, n.s. = not significant). Black dots represent biological replicates. (**B**) Mitochondria-localized and cytosolic GFP fluorescence of stable HEK293 cell lines presenting low (HEK293/GFP#1), intermediate (HEK293/GFP#2) and high (HEK293/GFP#3) GFP expression levels transiently transfected with plasmid expressing VHH_MOM_ (450 ng). Mitochondria-localized GFP fluorescence intensity values were obtained by quantifying the GFP signal that co-localizes with the MitoTracker stain. Data are reported as mean ± s.e.m. (*n* = 3, *P *<* *0.05, Student’s *t*-test). Black dots represent the biological replicates.

To test the extent to which the VHH_Loc_ expression level affects GFP localization, we monitored GFP localization within a representative subcellular compartment (mitochondria) upon modulation of the corresponding VHH_Loc_ variant (VHH_MOM_) expression level. Specifically, HEK293/GFP#1 cells were transiently transfected with varying amounts of VHH_MOM_ expressing plasmid (10–450 ng) and the extent of colocalization of GFP and MitoTracker stain quantified as a function of plasmid amount. Transfected cells were selected and analyzed based on expression of the transfection control (iRFP). The GFP signal of transfected cells was quantified (78). Localization of GFP to the mitochondria was found to depend on the VHH_MOM_ expression level until a saturation point. Specifically, the extent of mitochondria-localized GFP in HEK293/GFP#1 cells increased exponentially (20–150 ng of plasmid) until a 16.5-fold maximum compared to cells not expressing VHH_MOM_ and plateaued upon transfection with higher amounts of VHH_MOM_ expressing plasmid (Figure 4A and Supplementary Figure S3A). The amount of cytoplasmic GFP was found not to vary dramatically under these conditions.

To further characterize VHH_Loc_-mediated localization, we investigated the effect of GFP expression level on GFP localization by quantifying mitochondria-localized GFP fluorescence in cells presenting different GFP expression levels. We used three stable HEK293 cell lines expressing different GFP levels, namely HEK293/GFP#1 (low), HEK293/GFP#2 (intermediate) and HEK293/GFP#3 (high). Specifically, HEK293/GFP#2 cells displayed a 6.3-fold increase in total GFP fluorescence signal compared to HEK293/GFP#1 cells and HEK293/GFP#3 cells displayed a 15.4-fold increase compared to HEK293/GFP#1 cells (Supplementary Figure S3B). These stable GFP-expressing cell lines were transiently transfected with the amount of VHH_MOM_ expressing plasmid (450 ng, Figure 4A) expected to maximize GFP localization. Transfected cells were analyzed by confocal microscopy to evaluate the amount of cytoplasmic GFP and the extent of GFP localization to the mitochondria (Figure 4B). These analyses revealed that GFP localization to the mitochondria depends on the total level of GFP. Specifically, mitochondria-localized GFP fluorescence of HEK293/GFP#2 and HEK293/GFP#3 cells was 2.8- and 6.7-fold compared to that of HEK293/GFP#1 cells reflecting the differences in total GFP levels (Figure 4B).

These results, taken together, demonstrate that the extent of localization of a cytosolic target protein to a specific subcellular compartment can be tuned using the NanoLoc platform by modulating the expression level of the VHH_Loc_ variant relative to the expression level of the target protein.

**Figure 5.**
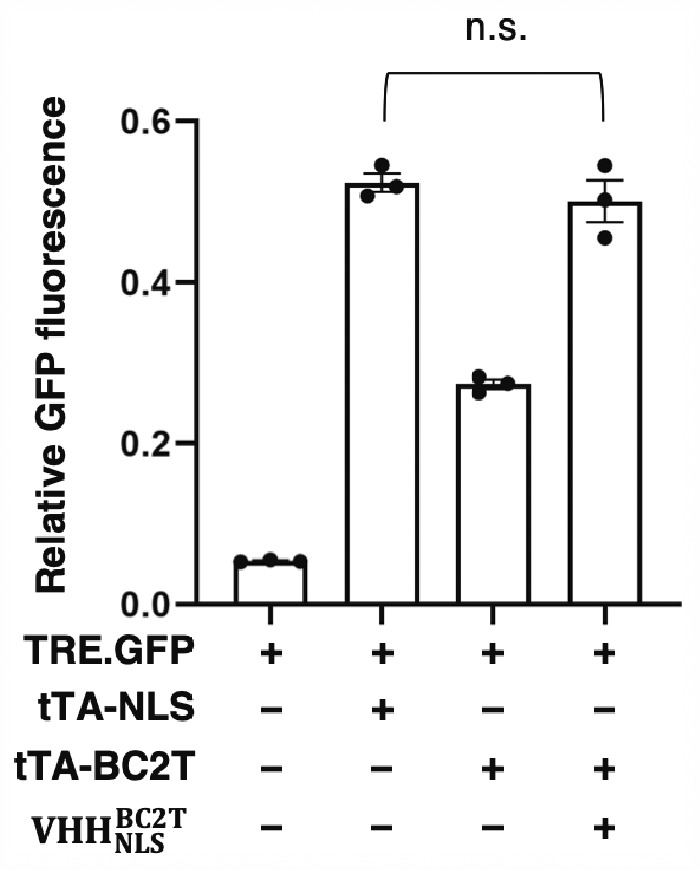
NanoLoc-mediated control of tTA function. GFP expression in HEK293 cells expressing *GFP* under the control of tTA (TRE.GFP) and either tTA-NLS or tTA-BC2T and ${\mathrm{VHH}}_{\mathrm{NLS}}^{\mathrm{BC}2T}$. Relative GFP fluorescence values were obtained by normalizing GFP signal to iRFP signal to correct for differences in transfection efficiency. Data are reported as mean ± s.e.m. (*n* = 3, *P *<* *0.01, Student’s *t*-test, n.s. = not significant). Black dots represent biological replicates.

To explore the functional effect of VHH_Loc_-mediated localization, we tested the use of the NanoLoc system to control the function of the Tc transactivator (tTA) (79). Because expression of a target gene under control of the tTA-inducible promoter depends on tTA nuclear localization, we explored the functional effect of VHH_Loc_-mediated localization by monitoring the expression of a reporter gene upon co-expression of a tTA-targeting VHH fused to a nuclear localization signal (VHH_NLS_). VHH_Loc_-mediated tTA localization was achieved using a tTA variant fused to the BC2 tag and a nanobody against the BC2 peptide tag (65) fused to the nuclear localization signal. VHH_Loc_-mediated tTA localization was compared to tTA nuclear localization achieved through expression of a tTA variant directly fused to a nuclear localization signal (80, 81). HEK293 cells were transfected with a plasmid encoding *GFP* under control of a cassette comprising 7 Tc operator repeats and the CMV promoter (79). The resulting cells were transfected with a plasmid encoding tTA fused to the BC2 tag (tTA-BC2T) and the BC2 tag-specific nanobody fused to the nuclear localization signal (${\mathrm{VHH}}_{\mathrm{NLS}}^{\mathrm{BC}2T}$) or with a plasmid encoding tTA fused to the nuclear localization signal (tTA-NLS). Cells transfected for the expression tTA-NLS presented an 8-fold increase in GFP fluorescence compared to control cells lacking tTA (Figure 5). Cells expressing tTA fused to the peptide tag (without any means of controlling tTA nuclear localization) presented about 50% of the GFP fluorescence signal detected in cells expressing tTA-NLS. Expression of the tag-specific nuclear NanoLoc (${\mathrm{VHH}}_{\mathrm{NLS}}^{\mathrm{BC}2T}$) restored GFP expression to a level comparable to that obtained in cells expressing the tTA-NLS (Figure 5). In addition to demonstrating the use of the NanoLoc to control localization-dependent protein function, these results also validate the use of the NanoLoc for controlling the localization of different types of target proteins.

**Figure 6.**
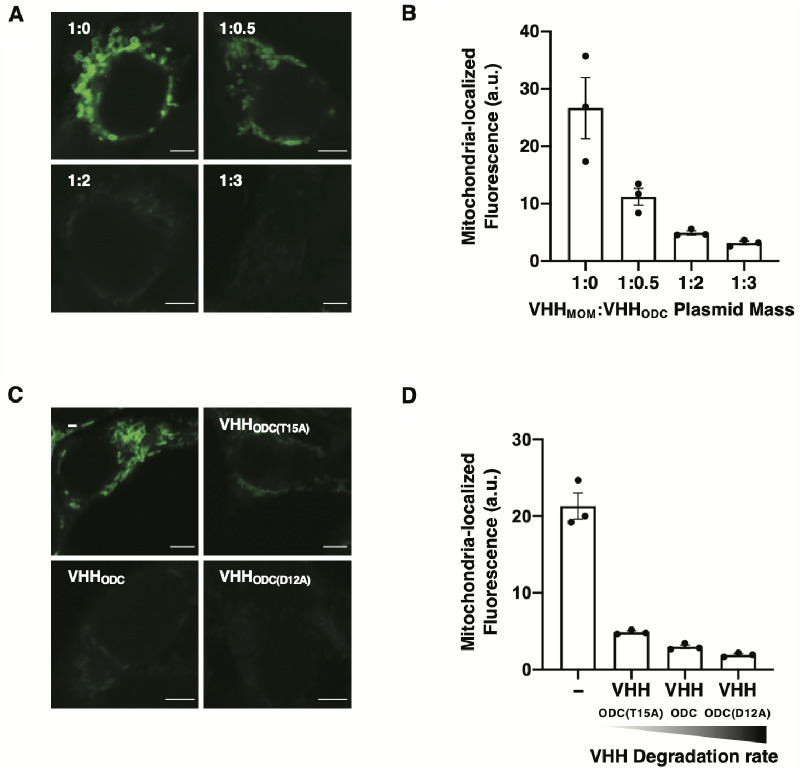
Modulation of mitochondria-localized GFP fluorescence via nanobody-mediated GFP degradation. (**A** and **B**) Confocal microscopy analyses of HEK293/GFP#1 cells transiently transfected for the expression of VHH_MOM_ and VHH_ODC_ using VHH_MOM_:VHH_ODC_ plasmid mass ratios of 1:0, 1:0.5, 1:2 and 1:3. A filler plasmid lacking *vhh* was used to maintain constant total plasmid mass. Images were analyzed 72 h post-transfection. (A) Representative images of mitochondria-localized GFP fluorescence. Scale bars: 5 µm. Brightness levels were adjusted and images of cells treated the same were subjected to the same adjustment. (B) Average mitochondria-localized GFP fluorescence. Fluorescence intensity values were obtained by quantifying the GFP signal that co-localizes with the MitoTracker stain. Data are reported as mean ± s.e.m. (*n* = 3, *P *<* *0.005, Student’s *t*-test). Black dots represent biological replicates. (**C** and **D**) Confocal microscopy analyses of HEK293/GFP#1 cells transiently transfected with a plasmid expressing VHH_MOM_ and a plasmid expressing a VHH_ODC_ variant (VHH_ODC(T15A)_, half-life 2.1 h; VHH_ODC_, half-life 1.3 h; and VHH_ODC(D12A)_, half-life 0.9 h) or lacking *vhh* in a 1:2 plasmid mass ratio and analyzed 72 h post-transfection. (C) Representative images of mitochondria-localized GFP fluorescence. Scale bars: 5 µm. Brightness levels were adjusted and images of cells treated the same were subjected to the same adjustment. (D) Average mitochondria-localized GFP fluorescence. Fluorescence intensity values were obtained by quantifying the GFP signal that co-localizes with the MitoTracker stain. Data are reported as mean ± s.e.m. (*n* = 3, *P *<* *0.005, Student’s *t*-test). Black dots represent biological replicates.

To investigate whether the extent of target protein subcellular localization could be further modulated post-translationally by controlling the target protein degradation rate, we combined nanobody-mediated modulation of GFP subcellular localization and degradation rate. GFP degradation rate was controlled using the previously developed NanoDeg platform consisting of target-specific nanobody variants fused to a degradation tag (VHH_Deg_) (66). Target localization (VHH_Loc_) and degradation (VHH_Deg_) were achieved using the same nanobody to ensure nanobody binding with the same affinity. HEK293/GFP#1 cells were transiently transfected for the expression of VHH_MOM_ and varying amounts of a VHH_Deg_ variant containing the 37 amino acid carboxy-terminal sequence of ornithine decarboxylase (VHH_ODC_) (82). Transfected cells were analyzed by confocal microscopy to quantify mitochondria-localized GFP fluorescence. Co-expression of VHH_MOM_ and VHH_ODC_ resulted in a reduction in mitochondria-localized GFP fluorescence compared to control cells lacking VHH_ODC_, which was proportional to the amount of VHH_ODC_-encoding plasmid (Figure 6A). Localized GFP fluorescence was reduced by 66%, 81% and 92% in cells expressing VHH_MOM_ to VHH_ODC_ ratios of 1:0.5, 1:2 and 1:3, respectively, compared to control cells (Figure 6B), indicating that the GFP levels in the compartment specified by the VHH_Loc_ can be controlled by modulating the expression of VHH_Deg_.

To investigate whether GFP subcellular localization could be further tuned by modulating the VHH_Deg_ degradation rate, rather than its expression level, we tested GFP localization upon expression of VHH_ODC_ variants presenting different half-lives. We used a set of VHH_ODC_ variants containing mutations in the ODC tag (83) that alter VHH_ODC_ half-life (66), namely VHH_ODC(T15A)_, VHH_ODC_ and VHH_ODC(D12A)_, which were reported to present half-lives of 2.1, 1.3 and 0.9 h, respectively (66). HEK293/GFP#1 cells were transiently transfected for the expression of VHH_MOM_ and VHH_ODC(T15A)_, VHH_ODC_ or VHH_ODC(D12A)_ and analyzed by confocal microscopy to quantify mitochondria-localized GFP fluorescence. Imaging analyses revealed a reduction in mitochondria-localized GFP fluorescence compared to control cells that was proportional to the VHH_ODC_ variant half-life. Localized GFP fluorescence was reduced by 67%, 80% and 87% in cells expressing VHH_ODC(T15A)_, VHH_ODC_ and VHH_ODC(D12A)_, respectively, compared to control cells (Figure 6C and D), indicating that GFP levels in the compartment specified by the VHH_Loc_ can be controlled by modulating the half-life of VHH_Deg_.

These results support the development of an integrated framework based on the NanoLoc and NanoDeg platforms for modulating target protein concentration in specific subcellular compartments by tuning nanobody-mediated target localization and nanobody-mediated target degradation.

### 3.2 Tuning GFP localization by modulating nanobody-mediated control of GFP localization and degradation

**Figure 7.**
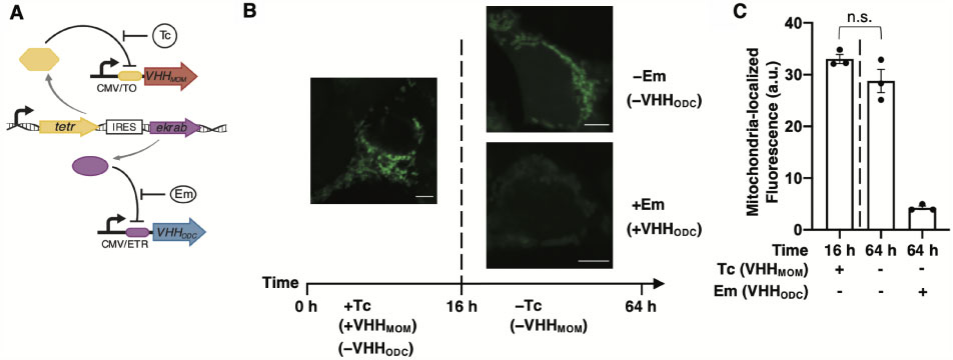
A dual-input expression system for the temporal control of GFP localization. (**A**) Schematic representation of the dual-input system to control expression of two VHH variants. The Tc repressor (TetR) and Em-dependent transrepressor (EKRAB) are constitutively expressed from a single promoter. Expression of VHH_MOM_ is repressed by TetR binding to the Tc operator (TO) and induced with Tc. Expression of VHH_ODC_ is repressed by EKRAB binding to the Em operator (ETR) and induced with Em. (**B** and **C**) Confocal microscopy analyses of HEK293/GFP2R cells transiently transfected with the dual expression systems described in (A) and cultured in the presence of Tc (50 ng/ml) for the first 16 h and then in the presence or absence of Em (500 ng/ml) for an additional 48 h. (B) Representative images of cells at 16 h (Tc treatment, left) and 64 h (right, Em-free media, top; Em-supplemented media, bottom). Scale bars: 5 µm. Brightness levels were adjusted and images of cells treated the same were subjected to the same adjustment. (C) Average mitochondria-localized GFP fluorescence of cells analyzed at 16 h (left of dashed line) and 64 h (right of dashed line) post-transfection. Fluorescence intensity values were obtained by quantifying the GFP signal that co-localizes with the MitoTracker stain. Data are reported as mean ± s.e.m. (*n* = 3, *P *<* *0.01, Student’s *t*-test). Black dots represent biological replicates.

To investigate strategies to use the NanoLoc and NanoDeg platforms for achieving temporal control of the target protein localization, we built a system to control the expression of multiple VHH variants. As proof-of-principle, we built the simplest configuration for the transcriptional control of multiple VHH variants, consisting of a dual-input system in which the expression of two VHH variants is regulated by inducible promoters. Specifically, we designed a system in which the expression of the two VHH variants is independently controlled by the Tc repressor (TetR) (84) and Em-dependent transrepressor (EKRAB) and can thus be independently regulated using the small-molecule inducers Tc and Em, respectively (Figure 7A). To build this system, we first transduced HEK293/GFP#1 cells for the expression of TetR and EKRAB and generated a stable cell line (HEK293/GFP2R) expressing GFP, TetR and EKRAB. Induction of gene expression was characterized by transiently transfecting HEK293/GFP2R cells for the expression of iRFP under the control of either the EKRAB or the TetR operator. Transfected cells were treated with the corresponding inducer and analyzed by flow cytometry to define the transgene expression levels as a function of inducer concentration (Supplementary Figure S4). The expression of VHH variants in the dual-input system regulated by EKRAB or TetR is expected to be comparable to that of iRFP under the same conditions.

To test whether the extent of target protein localization to an extracytosolic compartment can be modulated by controlling the target degradation rate after induction of subcellular localization, we explored the use of a VHH_Loc_ to first induce target subcellular localization followed by the expression of a VHH_Deg_ to control target degradation rate (Figure 7A). Specifically, we implemented the dual-input system by transiently transfecting HEK293/GFP2R cells for the expression of VHH_MOM_ under control of the Tc-inducible operator and the expression of VHH_ODC_ under control of the Em-inducible operator. Transfected cells were first treated with Tc to induce the expression of VHH_MOM_. The resulting sample presenting localization of GFP to the mitochondria (Figure 7B, left) was cultured in the absence of Tc to discontinue the expression of VHH_MOM_ and evaluate the residence time of GFP in the mitochondria (Figure 7B, top right). A sample of cells cultured in the absence of Tc was also exposed to Em to induce expression of VHH_ODC_ and evaluate whether VHH_ODC_-mediated degradation results in depletion of mitochondrial GFP (Figure 7B, bottom right). Confocal microscopy analyses of mitochondria-localized GFP fluorescence revealed that cells not exposed to Em and thus not expressing VHH_ODC_ present minimal decrease in mitochondria-localized GFP fluorescence compared to cells analyzed at the end of the Tc induction period. Induction of VHH_ODC_ via cell treatment with Em, on the other hand, resulted in 87% decrease in mitochondria-localized GFP fluorescence (Figure 7C). These results demonstrate the residence time of a target protein localized in a subcellular compartment can be modulated by controlling proteasome-mediated degradation of the target protein.

**Figure 8.**
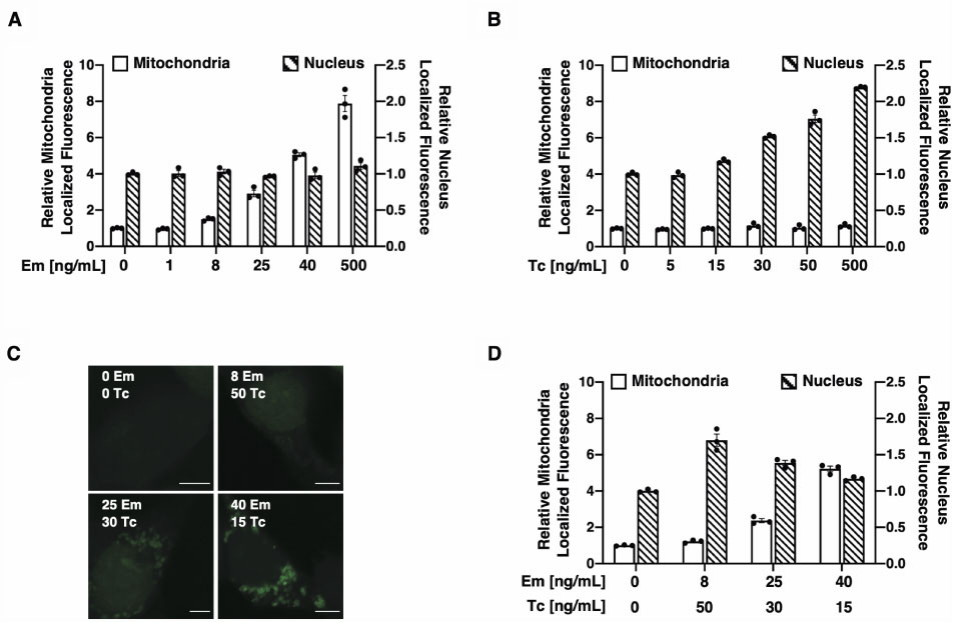
Modulation of GFP localization to different subcellular compartment using a dual-input nanobody expression system. (**A**–**D**) Mitochondria- and nucleus-localized GFP fluorescence in HEK293/GFP2R cells transiently transfected for the expression of VHH_MOM_ under control of the Em-inducible operator and VHH_NLS_ under control of the Tc-inducible operator, treated with the inducers (Em and Tc) 16 h post-transfection and analyzed after 48 h of induction. (A) GFP fluorescence localized with the mitochondria (white bars) and with the nucleus (striped bars) in transfected cells cultured in the presence of Em (0–500 ng/ml) and in the absence of Tc. Relative fluorescence values were obtained by normalizing the GFP signal that co-localizes with the VHH_Loc_-specific compartment to the co-localized GFP signal of untreated samples. (B) GFP fluorescence localized with the mitochondria (white bars) and with the nucleus (striped bars) in transfected cells cultured in the presence of Tc (0–500 ng/ml) and in the absence of Em. Relative fluorescence values were obtained as described in (A). (C) Representative images of cells treated with Tc and Em. Scale bars: 5 µm. Brightness levels were adjusted and images of cells treated the same were subjected to the same adjustment. (D) GFP fluorescence localized with the mitochondria (white bars) and with the nucleus (striped bars) in transfected cells cultured in the presence of both Em and Tc. Relative fluorescence values were obtained as described in (A). All data are reported as mean ± s.e.m. (*n* = 3, *P *<* *0.05, Student’s *t*-test). Black dots represent biological replicates.

To test whether the extent of target protein localization to distinct extracytosolic compartments can by modulated by controlling the expression level of the corresponding VHH_Loc_ variants, we monitored GFP signal in two subcellular compartments (mitochondria and nucleus) upon modulation of the expression level of two VHH_Loc_ variants (VHH_MOM_ and VHH_NLS_). Specifically, we used the dual-input system to induce expression of VHH_MOM_ under the control of the Em-inducible operator and VHH_NLS_ under the control of the Tc-inducible operator. Cells were treated with Em and Tc, separately or in combination, at concentrations expected to result in linear changes in target gene expression as estimated from iRFP induction analyses (Supplementary Figure S4). Localization of GFP to the mitochondria and nuclei was assessed using confocal microscopy. Cell treatment with Em resulted in an increase in mitochondria-localized GFP fluorescence to a 7.9-fold maximum compared to untreated cells and did not affect localization of GFP to the nucleus (Figure 8A). Cell treatment with Tc resulted in an increase in nucleus-localized GFP fluorescence to a 2.2-fold maximum compared to untreated cells and did not affect localization of GFP to the mitochondria (Figure 8B). Cells exposed to Em and Tc displayed GFP localization to both the nucleus and mitochondria (Figure 8C). The extent of GFP colocalization with the nucleus and the mitochondria depended on the concentration of Em and Tc and thus on the expression level of the compartment-specific VHH_Loc_ variants (Figure 8D). These results demonstrate that localization of a target protein can be quantitatively tuned between different subcellular compartments by modulating the expression of the appropriate VHH_Loc_ variants.

### 3.3 Design of genetic toggle switch for spatial control of GFP output

**Figure 9.**
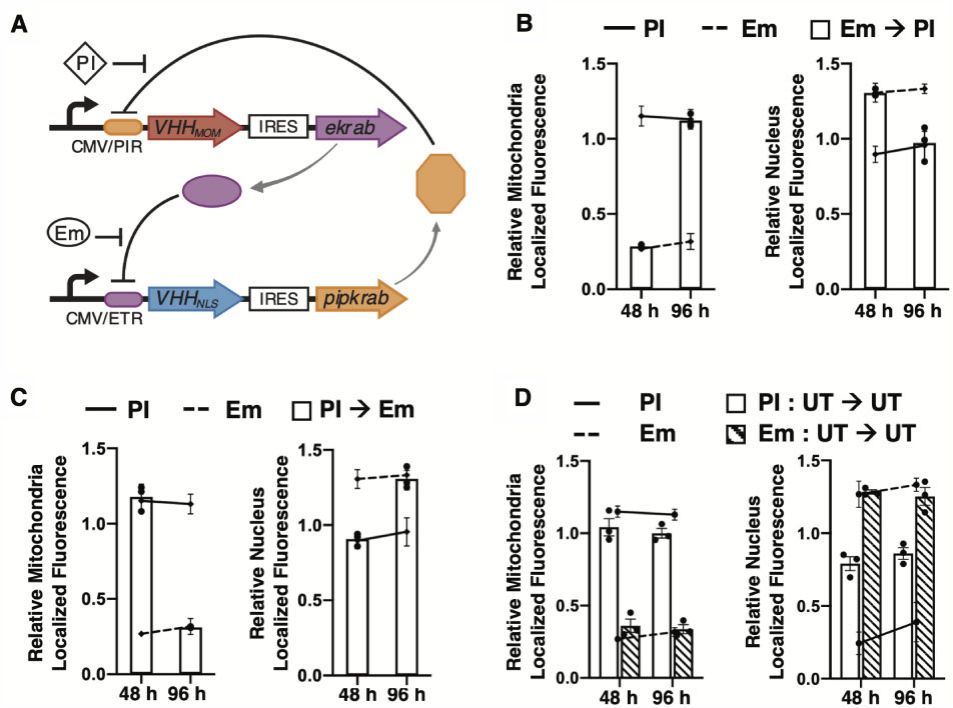
A synthetic toggle switch to control GFP subcellular localization. (**A**) Schematic representation of the spatial toggle switch. Expression of VHH_MOM_ is linked to that of EKRAB and expression of VHH_NLS_ is linked to that of PIPKRAB. Expression of VHH_MOM_ and EKRAB is repressed by PIPKRAB binding to the pristinamycin operator (PIR) and induced by PI. Expression of VHH_NLS_ and PIPKRAB is repressed by EKRAB binding to the Em operator (ETR) and induced by Em. (**B**–**D**) Mitochondria- and nucleus-localized GFP fluorescence of HEK293/GFP#1 cells transiently transfected with plasmids for expression of the spatial toggle switch described in A and analyzed by confocal microscopy. Relative fluorescence values were obtained by normalizing the GFP signal that co-localizes with the VHH_Loc_-specific compartment to the co-localized GFP signal of untreated samples. (B) Relative localized GFP fluorescence of transfected cells cultured in the presence of PI during transfection to initialize cells to VHH_MOM_ and PIPKRAB expression and then treated with Em (500 ng/ml) for the first 48 h, and with PI (500 ng/ml) for the other 48 h. Cells were analyzed at the end of Em treatment (48 h) and at the end of the PI treatment (96 h). Control cells were treated continuously with PI (solid line) and Em (dashed line). (C) Relative localized GFP fluorescence of transfected cells cultured in the presence of Em during transfection to initialize cells to VHH_NLS_ and EKRAB expression and then treated with PI (500 ng/ml) for the first 48 h, and with Em (500 ng/ml) for other 48 h. Cells were analyzed at the end of PI treatment (48 h) and at the end of Em treatment (96 h). Control cells were treated continuously with PI (solid line) and Em (dashed line). (D) Relative localized GFP fluorescence of transfected cells cultured in the presence of PI (white bars) or of Em (striped bars) during transfection (16 h) and then in inducer-free medium (untreated, UT) for 96 h. Cells were analyzed 48 and 96 h after removal of the inducer. Control cells were treated continuously with PI (solid line) and Em (dashed line). All data are reported as mean ± s.e.m. (*n* = 3, *P *<* *0.05, Student’s *t*-test). Black dots represent biological replicates.

To investigate whether the NanoLoc can be integrated within larger genetic networks to control the circuit components spatially, we built a genetic toggle switch circuit that regulates expression of two VHH_Loc_ variants such that the state of the toggle switch dictates the expression of each VHH_Loc_ variant and the resulting subcellular localization of the target protein. Toggle switch topologies in bacteria (85–87) and mammalian cells (69, 88, 89) are typically based on the function of two transcriptional repressors that mutually repress each other. To generate a toggle switch for the spatial control of GFP localization between two subcellular compartments, we modified the classic toggle switch configuration (69, 85) to link the expression of two VHH_Loc_ variants to that of the two repressors through an internal ribosome entry site (IRES) sequence (Figure 9A). Specifically, HEK293/GFP#1 cells were transiently transfected for the expression of VHH_MOM_ and EKRAB under the control of the pristinamycin I (PI)-inducible operator and VHH_NLS_ and PIPKRAB under the control of the Em-inducible operator. The same IRES was used to link the expression of VHH_MOM_ and VHH_NLS_ to that of EKRAB and PIPKRAB to ensure equal ratios of expression. Transfected cells were treated with Em and PI and imaged by confocal microscopy to quantify GFP localization and verify that the state of the toggle switch determines the target’s subcellular localization.

To investigate the use of the spatial toggle switch to shift the localization of GFP from the nucleus to the mitochondria, transfected HEK293/GFP#1 cells expressing the VHH_Loc_-modified toggle switch were treated with pulses of Em and PI. Specifically, cells were treated with PI during transfection to initialize the toggle to mitochondrial localization by inducing the expression of VHH_MOM_ and PIPKRAB. Transfected cells were first treated with Em for 48 h to induce the expression of VHH_NLS_ (and EKRAB), resulting in the repression of VHH_MOM_ (and PIPKRAB), and then with PI for an additional 48 h to induce the expression of VHH_MOM_ (and PIPKRAB), resulting in the repression of VHH_NLS_ (and EKRAB). Control cells treated continuously with Em or PI to maximize the expression of VHH_NLS_ or VHH_MOM_, respectively, provided measurements of maximum localization of GFP to the mitochondria (PI treatment, Figure 9B–D solid lines) or the nucleus (Em treatment, Figure 9B–D, dashed lines) in the system. Imaging analyses at the end of the Em treatment (Figure 9B, 48 h) showed low mitochondrial localization (left) and high nuclear localization (right) and the extent of localization to both the mitochondria and the nucleus was comparable to that observed in cells treated continuously with Em. After the switch to PI treatment (Figure 9B, 96 h), cells displayed high mitochondrial localization (left) and low nuclear localization (right) and the extent of localization to both the mitochondria and the nucleus was comparable to that observed in cells treated continuously with PI (Figure 9B). These results indicate that GFP localization is switched from a state of predominantly nucleus-localized GFP to a state of predominantly mitochondria-localized GFP by switching from the expression of VHH_NLS_ to the expression of VHH_MOM_.

To demonstrate reversible switching of the toggle, we tested the opposite transition from mitochondria-localized GFP to nucleus-localized GFP. Cells were transiently transfected for the expression of the spatial toggle switch in the presence of Em to initialize the toggle to nuclear localization by inducing the expression of VHH_NLS_ and EKRAB. Transfected cells were first treated with PI for 48 h to induce the expression of VHH_MOM_ (and PIPKRAB), resulting in the repression of VHH_NLS_ (and EKRAB), and then with Em for an additional 48 h to induce the expression of VHH_NLS_ (and EKRAB), resulting in the repression of VHH_MOM_ (and PIPKRAB). Confocal microscopy analyses of GFP subcellular localization at the end of PI treatment (Figure 9C, 48 h) showed high mitochondrial localization (left) and low nuclear localization (right). Similar to the previous experiment, the extent of localization to both the mitochondria and the nucleus was comparable to that observed in cells treated continuously with PI. After the switch to Em treatment (Figure 9C, 96 h), cells displayed low mitochondrial localization (left) and high nuclear localization (right) at levels corresponding to those observed in cells treated continuously with Em (Figure 9C), indicating a shift from nuclear to mitochondrial localization upon switch of the expression of VHH_Loc_.

These results (Figure 9B and C), taken together, indicate that localization of the target protein can be reversibly switched between the mitochondria and the nucleus by switching the state of the spatial toggle switch circuit.

In addition to reversible switching, another key feature of the toggle switch topology is bistability of the system (69, 85). Bistability is denoted by the presence of two path-dependent stable output states for given inputs (i.e. a fixed set of inducer concentrations) (90). To demonstrate bistability of the spatial toggle switch, we treated cells with two different inducers to generate two different initial conditions and then removed the inducers to subject cells exposed to different initial conditions to a common input condition. We monitored GFP localization to determine whether the outputs associated with each initial condition remained stable. Specifically, HEK293/GFP#1 cells were exposed to a pulse of either PI or EM during transient transfection for expression of the spatial toggle switch and then incubated without inducer. Confocal microscopy analyses of GFP subcellular localization 48 and 96 h after the removal of PI (Figure 9D, white bars) showed high mitochondrial localization (left) and low nuclear localization (right). The extent of localization to both the mitochondria and the nucleus was again comparable to that observed in cells induced with PI and cultured continuously in the presence of PI for 96 h (Figure 9D, solid line). Imaging analyses of cells treated with a pulse of Em (Figure 9D, striped bars) displayed low mitochondrial localization (left) and high nuclear localization (right) at levels comparable to those observed in cells treated continuously with Em (dashed line). The GFP localization levels achieved upon cell exposure to a pulse of inducer were maintained for 96 h post-induction, indicating the spatial toggle switch displays bistable localization to either the mitochondria or the nucleus depending on the initial conditions of the circuit.

These results indicate that the NanoLoc platform can be integrated with a genetic circuit to exert spatial control over the outputs through appropriate design of the circuit topology, thus providing an additional mode for control for the design of synthetic gene networks.

## 4. Discussion

Strategies for user-defined control of protein subcellular localization allow investigating the role of protein localization in a variety of cellular function (15, 17, 24, 91), including mechanisms that mediate the cellular response to external stimuli (17–19, 22). An array of intrinsic localization signals that dictate the subcellular address of proteins has been characterized (13). Fusion of localization signals to target proteins enables protein relocation to the desired subcellular compartment (51) but requires genetic manipulation of the target protein, which may affect the protein’s native function independently of localization (52). Most importantly, direct fusion of target proteins to localization signals does not allow modulating the extent of target localization to the desired subcellular compartment. Interacting protein domains have also been employed to engineer inducible localization systems in which the target and a compartment-specific protein are fused to interacting partners (40, 41, 50, 56–61). While providing an opportunity for tunable and reversible control of protein localization, these systems depend largely on the choice of binding partner and often require extensive development and re-engineering for customization to other target proteins. Regulating target localization through the use of target-specific recognition molecules functionalized with localization signals enables spatial control of the resulting complex without the need for target manipulation (63, 64, 92, 93). Nanobody-mediated localization has enabled a number of fundamental studies, including investigations of the role of fascin and cortactin in invadopodium formation (64) and the role of survivin localization during mitosis (63). These methods, however, do not provide tunable control over the extent of target localization and typically result in irreversible localization. The potential of these strategies for the development of a universal, plug-and-play technology for reversible control of target localization that can be adapted to any cellular protein and used to direct localization to different subcellular compartments remains uncharacterized.

In this study, we addressed the need for a technology for controlling localization of cellular proteins with multi-compartment specificity, tunable control over the localized target concentration, and dynamic control over the target residence time. We developed a modular system (NanoLoc) based on nanobodies, which are the smallest antigen recognition domain and bind to cellular targets with high specificity and selectivity (94–96), and a comprehensive series of localization signal sequences, which when fused to the target-specific nanobody determine localization of the nanobody-target protein complex. Nanobodies are readily obtained from immunized Camelids (97–102) and can be evolved (103–108) or engineered (109) to target a seemingly unlimited number of sequences using display technologies commonly employed to evolve mammalian antibodies. The small size and stability of nanobodies allows for easy expression (110, 111) and excellent tissue penetration (112). The NanoLoc platform can be adapted to achieve control of a target protein of interest through the use of a target-specific nanobody. We thus envision that this platform could be customized to target virtually any cellular protein sequence, as well as protein conformation (113–115) and post-translational modification (113, 116). While possibly not generalizable to some classes of target proteins for which stability and function is typically contingent upon interaction with cellular structures such as membranes, this approach is expected to present great potential for controlling the function of soluble proteins. It is also important to note that, the extent of localization of the target protein using the NanoLoc platform may vary depending on the cellular context due to cell type specific variability (117).

Customization of the nanobody-based platform developed in this study with respect to the localization determining sequence allows user-defined control of target localization to potentially any subcellular compartment (13). The choice of localization signal also determines the target residence time within the signal-specific compartment. The molecular mechanisms determining the target residence time within the signal-specific compartments remains uncharacterized. Understanding the response kinetics of the VHH_Loc_ variants would inform the design of NanoLoc systems with superior control over the kinetics of target subcellular localization. The reversible nature of the VHH_Loc_-induced perturbation allows minimizing irreversible downstream effects and exposing phenotypic changes that depend on the duration of the perturbation. Such localization-based perturbation (40) may thus prove useful in elucidating mechanisms regulating cell signaling (118, 119) and viral infections (92, 120, 121). The NanoLoc platform could also be used to investigate the effect of aberrant localization of disease-related proteins (39, 122, 123). Finally, the NanoLoc platform could provide a useful tool to control localization of multiple cellular proteins simultaneously; such applications would require optimization of the target-specific nanobody expression levels to account for differences in nanobody-target binding affinities.

We demonstrated that our nanobody-based system can achieve spatial and temporal control over a target protein by combining nanobody-mediated localization (NanoLoc) and degradation (NanoDeg) technologies with transcriptional regulation. Small-molecule inducible promoters were used to drive orthogonal expression of VHH_Loc_ and VHH_Deg_ variants in a dual-input system, thereby tuning the duration of localization and the localized concentration of a target protein in multiple subcellular compartments. The NanoLoc system could also be expanded to localize multiple proteins to a single subcellular compartment by combining distinct VHH_Loc_ variants customized to recognize multiple targets. Developing the NanoLoc platform to control multiple targets in a distinct cellular compartment could open the way to a variety of metabolic engineering applications as it provides a powerful approach to regulate metabolite fluxes by controlling the local concentration of key reaction components (44, 124).

Finally, we demonstrated the use of the GFP-specific NanoLoc as a modular unit for synthetic genetic circuits that allows spatial control of the circuit’s reporter protein. Synthetic mammalian gene networks are typically designed to regulate the concentration of a single reporter protein (125). By contrast, the NanoLoc enables regulation of both location and local concentration of the reporter protein, thereby broadening the cell engineering potential of synthetic gene networks. We integrated VHH_Loc_ variants within a transcriptional toggle switch configuration and demonstrated reversible localization of a target protein that depends on the state of the toggle switch. We anticipate that this strategy for spatial control of genetic circuit outputs will provide a useful method for orthogonal regulation of spatially constrained reactions using synthetic circuits that can reversibly locate a key reaction component (25) in response to the state of the genetic circuit.

In summary, the NanoLoc system provides an innovative technology for spatial and temporal control of protein concentration in mammalian cells that can be customized for controlling the localization of target cellular proteins and integrated within more complex transcription-based circuitries to diversify the functionality of synthetic gene circuits.

## Supplementary data

Supplementary Data are available at SYNBIO Online.

## Author contributions

B.J. and L.S. designed the project. B.J. and B.B. constructed plasmids and cell lines. B.J., B.B. and Z.W. performed experiments. B.J. and B.B. analyzed the data. S.M.L. contributed to the image analysis. B.J. and L.S. wrote the article. B.B., B.J. and L.S. revised the manuscript.

## Supplementary Material

ysab002_Supplementary_Data

## References

[1] Berg J. , Tymoczko J., Stryer L. (2002) Biochemistry, 5th edn. W. H. Freeman and Company, New York.

[2] Bauer N.C. , Doetsch P.W., Corbett A.H. (2015) Mechanisms regulating protein localization. Traffic, 16, 1039–1061. 26172624 10.1111/tra.12310

[3] Coulon A. , Chow C.C., Singer R.H., Larson D.R. (2013) Eukaryotic transcriptional dynamics: from single molecules to cell populations. Nat. Rev. Genet., 14, 572–584. 23835438 10.1038/nrg3484 PMC3807637

[4] Weake V.M. , Workman J.L. (2010) Inducible gene expression: diverse regulatory mechanisms. Nat. Rev. Genet., 11, 426–437. [10.1038/nrg2781]. 20421872 10.1038/nrg2781

[5] Lambert S.A. , Jolma A., Campitelli L.F., Das P.K., Yin Y., Albu M., Chen X., Taipale J., Hughes T.R., Weirauch M.T. (2018) The human transcription factors. Cell, 172, 650–665. 29425488 10.1016/j.cell.2018.01.029 PMC12908702

[6] Lelli K.M. , Slattery M., Mann R.S. (2012) Disentangling the many layers of eukaryotic transcriptional regulation. Annu. Rev. Genet., 46, 43–68. 22934649 10.1146/annurev-genet-110711-155437 PMC4295906

[7] Hochstrasser M. (1995) Ubiquitin, proteasomes, and the regulation of intracellular protein degradation. Curr. Opin. Cell Biol., 7, 215–223. 7612274 10.1016/0955-0674(95)80031-x

[8] Seglen P.O. , Bohley P. (1992) Autophagy and other vacuolar protein degradation mechanisms. Experientia, 48, 158–172. [10.1007/BF01923509] 1740188 10.1007/BF01923509

[9] Goldberg A.L. (2003) Protein degradation and protection against misfolded or damaged proteins. Nature, 426, 895–899. 14685250 10.1038/nature02263

[10] Liu H. , Urbé S., Clague M.J. (2012) Selective protein degradation in cell signalling. Semin. Cell Dev. Biol., 23, 509–514. 22343089 10.1016/j.semcdb.2012.01.014

[11] Kraft C. , Peter M., Hofmann K. (2010) Selective autophagy: ubiquitin-mediated recognition and beyond. Nat. Cell Biol., 12, 836–841. 20811356 10.1038/ncb0910-836

[12] Varshavsky A. (2011) The N-end rule pathway and regulation by proteolysis. Protein Sci., 20, 1298–1345. [10.1002/pro.666]. 21633985 10.1002/pro.666 PMC3189519

[13] Negi S. , Pandey S., Srinivasan S.M., Mohammed A., Guda C. (2015) LocSigDB: a database of protein localization signals. Database, 2015, bav003–7. 25725059 10.1093/database/bav003 PMC4343182

[14] Chan P. , Lovrić J., Warwicker J. (2006) Subcellular pH and predicted pH-dependent features of proteins. Proteomics, 6, 3494–3501. 16705750 10.1002/pmic.200500534

[15] Sweetlove L.J. , Fernie A.R. (2013) The spatial organization of metabolism within the plant cell. Annu. Rev. Plant Biol., 64, 723–746. 23330793 10.1146/annurev-arplant-050312-120233

[16] Jakobson C.M. , Tullman-Ercek D., Mangan N.M. (2018) Spatially organizing biochemistry: choosing a strategy to translate synthetic biology to the factory. Sci. Rep., 8, 1–13. [10.1038/s41598-018-26399-0] 29844460 10.1038/s41598-018-26399-0 PMC5974357

[17] Kakkar P. , Singh B.K. (2007) Mitochondria: a hub of redox activities and cellular distress control. Mol. Cell. Biochem., 305, 235–253. [10.1007/s11010-007-9520-8] 17562131 10.1007/s11010-007-9520-8

[18] Good M.C. , Zalatan J.G., Lim W.A. (2011) Scaffold proteins: hubs for controlling the flow of cellular information. Science, 332, 680–686.21551057

[19] Morrison D.K. , Davis R.J. (2003) Regulation of MAP kinase signaling modules by scaffold proteins in mammals. Annu. Rev. Cell Dev. Biol., 19, 91–118. 14570565 10.1146/annurev.cellbio.19.111401.091942

[20] Konopka M.C. , Shkel I.A., Cayley S., Record M.T., Weisshaar J.C. (2006) Crowding and confinement effects on protein diffusion in vivo. J. Bacteriol., 188, 6115–6123. 16923878 10.1128/JB.01982-05 PMC1595386

[21] Ólafsson G. , Thorpe P.H. (2016) Synthetic physical interactions map kinetochore-checkpoint activation regions. G3, 6, 2531–2542. 27280788 10.1534/g3.116.031930 PMC4978906

[22] Takano A. , Usui I., Haruta T., Kawahara J., Uno T., Iwata M., Kobayashi M. (2001) Mammalian target of rapamycin pathway regulates insulin signaling via subcellular redistribution of insulin receptor substrate 1 and integrates nutritional signals and metabolic signals of insulin. Mol. Cell. Biol., 21, 5050–5062. 11438661 10.1128/MCB.21.15.5050-5062.2001 PMC87231

[23] Kamatkar S. , Radha V., Nambirajan S., Reddy R.S., Swarup G. (1996) Two splice variants of a tyrosine phosphatase differ in substrate specificity, DNA binding, and subcellular location. J. Biol. Chem., 271, 26755–26761. 8900155 10.1074/jbc.271.43.26755

[24] Jagnandan D. , Sessa W.C., Fulton D. (2005) Intracellular location regulates calcium-calmodulin-dependent activation of organelle-restricted eNOS. Am. J. Physiol. Cell Physiol., 289, C1024–C1033. [10.1152/ajpcell.00162.2005] 15917301 10.1152/ajpcell.00162.2005

[25] Hao N. , Budnik B.A., Gunawardena J., O'Shea E.K. (2013) Tunable signal processing through modular control of transcription factor translocation. Science, 339, 460–464. 23349292 10.1126/science.1227299 PMC3746486

[26] Jeong S.Y. , Seol D.W. (2008) The role of mitochondria in apoptosis. J. Biochem. Mol. Biol., 41, 011–022. 10.5483/bmbrep.2008.41.1.011 18304445

[27] Grant B.D. , Donaldson J.G. (2009) Pathways and mechanisms of endocytic recycling. Nat. Rev. Mol. Cell Biol., 10, 597–608. [10.1038/nrm2755] 19696797 10.1038/nrm2755 PMC3038567

[28] Ryu J. , Park S.H. (2015) Simple synthetic protein scaffolds can create adjustable artificial MAPK circuits in yeast and mammalian cells. Sci. Signal., 8, ra66. 26126717 10.1126/scisignal.aab3397

[29] Agapakis C.M. , Boyle P.M., Silver P.A. (2012) Natural strategies for the spatial optimization of metabolism in synthetic biology. Nat. Chem. Biol., 8, 527–535. [10.1038/nchembio.975] 22596204 10.1038/nchembio.975

[30] Chatterjee G. , Dalchau N., Muscat R.A., Phillips A., Seelig G. (2017) A spatially localized architecture for fast and modular DNA computing. Nat. Nanotechnol., 12, 920–927. [10.1038/nnano.2017.127] 28737747 10.1038/nnano.2017.127

[31] Yogurtcu O.N. , Johnson M.E. (2018) Cytosolic proteins can exploit membrane localization to trigger functional assembly. PLoS Comput. Biol., 14, 1–28. [10.1371/journal.pcbi.1006031] 10.1371/journal.pcbi.1006031 PMC5854442 29505559

[32] Myhrvold C. , Polka J.K., Silver P.A. (2016) Synthetic lipid-containing scaffolds enhance production by colocalizing enzymes. ACS Synth. Biol., 5, 1396–1403. 27487319 10.1021/acssynbio.6b00141

[33] Bassell G.J. , Kelic S. (2004) Binding proteins for mRNA localization and local translation, and their dysfunction in genetic neurological disease. Curr. Opin. Neurobiol., 14, 574–581. [10.1016/j.conb.2004.08.010] 15464890 10.1016/j.conb.2004.08.010

[34] Wang W. , Van Niekerk E., Willis D.E., Twiss J.L. (2007) RNA transport and localized protein synthesis in neurological disorders and neural repair. Dev. Neurobiol., 67, 1166–1182. [10.1002/dneu.20511] 17514714 10.1002/dneu.20511

[35] Kowall N.W. , Kosik K.S. (1987) Axonal disruption and aberrant localization of tau protein characterize the neuropil pathology of Alzheimer’s disease. Ann. Neurol., 22, 639–643. [10.1002/ana.410220514] 3122646 10.1002/ana.410220514

[36] Calvo S. , Jain M., Xie X., Sheth S.A., Chang B., Goldberger O.A., Spinazzola A., Zeviani M., Carr S.A., Mootha V.K. (2006) Systematic identification of human mitochondrial disease genes through integrative genomics. Nat. Genet., 46, 248–255. [10.1038/ng1776] 10.1038/ng1776 16582907

[37] Lee D.S. , Park J., Kay K.A., Christakis N.A., Oltvai Z.N., Barabási A.L. (2008) The implications of human metabolic network topology for disease comorbidity. Proc. Natl. Acad. Sci. USA, 105, 9880–9885. [10.1073/pnas.0802208105] 18599447 10.1073/pnas.0802208105 PMC2481357

[38] Wang X. , Li S. (2014) Protein mislocalization: mechanisms, functions and clinical applications in cancer. Biochim. Biophys. Acta Rev. Cancer, 1846, 13–25. [10.1016/j.bbcan.2014.03.006] 10.1016/j.bbcan.2014.03.006 PMC4141035 24709009

[39] Hung M.C. , Link W. (2011) Protein localization in disease and therapy. J. Cell Sci., 124, 3381–3392. [10.1242/jcs.089110] 22010196 10.1242/jcs.089110

[40] Robinson M.S. , Sahlender D.A., Foster S.D. (2010) Rapid inactivation of proteins by rapamycin-induced rerouting to mitochondria. Dev. Cell, 18, 324–331. 20159602 10.1016/j.devcel.2009.12.015 PMC2845799

[41] Umeda N. , Ueno T., Pohlmeyer C., Nagano T., Inoue T. (2011) A photocleavable rapamycin conjugate for spatiotemporal control of small GTPase activity. J. Am. Chem. Soc., 133, 12–14. 21142151 10.1021/ja108258d PMC3850177

[42] Toby G.G. , Golemis E.A. (2001) Targeting proteins to specific cellular compartments to optimize physiological activity. Methods Enzymol., 332, 77–87. 11305119 10.1016/s0076-6879(01)32193-6

[43] Yadav V.G. , De Mey M., Giaw Lim C., Kumaran Ajikumar P., Stephanopoulos G. (2012) The future of metabolic engineering and synthetic biology: towards a systematic practice. Metab. Eng., 14, 233–241. 22629571 10.1016/j.ymben.2012.02.001 PMC3615475

[44] Lee H. , DeLoache W.C., Dueber J.E. (2012) Spatial organization of enzymes for metabolic engineering. Metab. Eng., 14, 242–251. 21946160 10.1016/j.ymben.2011.09.003

[45] Prelich G. (2012) Gene overexpression: uses, mechanisms, and interpretation. Genetics, 190, 841–854. 22419077 10.1534/genetics.111.136911 PMC3296252

[46] Blackstock W.P. , Weir M.P. (1999) Proteomics: quantitative and physical mapping of cellular proteins. Trends Biotechnol., 17, 121–127. [10.1016/S0167-7799(98)01245-1] 10189717 10.1016/s0167-7799(98)01245-1

[47] Kim H. , Kim J.S. (2014) A guide to genome engineering with programmable nucleases. Nat. Rev. Genet., 15, 321–334. 24690881 10.1038/nrg3686

[48] Bashor C.J. , Horwitz A.A., Peisajovich S.G., Lim W.A. (2010) Rewiring cells: synthetic biology as a tool to interrogate the organizational principles of living systems. Annu. Rev. Biophys., 39, 515–537. 20192780 10.1146/annurev.biophys.050708.133652 PMC2965450

[49] Olson E.J. , Tabor J.J. (2012) Post-translational tools expand the scope of synthetic biology. Curr. Opin. Chem. Biol., 16, 300–306. 22766485 10.1016/j.cbpa.2012.06.003

[50] Harmansa S. , Alborelli I., Bieli D., Caussinus E., Affolter M. (2017) A nanobody-based toolset to investigate the role of protein localization and dispersal in Drosophila. eLife, 6, 1–22. 10.7554/eLife.22549 PMC5388529 28395731

[51] Goedhart J. , Von Stetten D., Noirclerc-Savoye M., Lelimousin M., Joosen L., Hink M.A., Van Weeren L., Gadella T.W.J., Royant A. (2012) Structure-guided evolution of cyan fluorescent proteins towards a quantum yield of 93%. Nat. Commun., 3, 1–9. [10.1038/ncomms1738] 10.1038/ncomms1738 PMC3316892 22434194

[52] Campbell,A E. , Bennett D. (2016) Targeting protein function: the expanding toolkit for conditional disruption. Biochem. J., 473, 2573–2589. [10.1042/BCJ20160240] 27574023 10.1042/BCJ20160240 PMC5003692

[53] Hosein R.E. , Williams S.A., Haye K., Gavin R.H. (2003) Expression of GFP-actin leads to failure of nuclear elongation and cytokinesis in Tetrahymena thermophila. J. Eukaryot. Microbiol., 50, 403–408. [10.1111/j.1550-7408.2003.tb00261.x] 14733431 10.1111/j.1550-7408.2003.tb00261.x

[54] Nelson M. , Silver P. (1989) Context affects nuclear protein localization in Saccharomyces cerevisiae. Mol. Cell. Biol., 9, 384–389. 2496300 10.1128/mcb.9.2.384-389.1989 PMC362612

[55] Douglas M.G. , McCammon M.T., Vassarotti A. (1986) Targeting proteins into mitochondria. Microbiol. Rev., 50, 166–178. 2941675 10.1128/mr.50.2.166-178.1986 PMC373062

[56] Putyrski M. , Schultz C. (2012) Protein translocation as a tool: the current rapamycin story. FEBS Lett., 586, 2097–2105. [10.1016/j.febslet.2012.04.061] 22584056 10.1016/j.febslet.2012.04.061

[57] Brown K.A. , Zou Y., Shirvanyants D., Zhang J., Samanta S., Mantravadi P.K., Dokholyan N.V., Deiters A. (2015) Light-cleavable rapamycin dimer as an optical trigger for protein dimerization. Chem. Commun., 51, 5702–5705. 10.1039/c4cc09442e 25716548

[58] Geda P. , Patury S., Ma J., Bharucha N., Dobry C.J., Lawson S.K., Gestwicki J.E., Kumar A. (2008) A small molecule-directed approach to control protein localization and function. Yeast, 25, 577–594. 18668531 10.1002/yea.1610

[59] Toettcher J.E. , Gong D., Lim W.A., Weiner O.D. (2011) Light control of plasma membrane recruitment using the Phy-PIF system. Methods Enzymol., 497, 409–423. [10.1016/B978-0-12-385075-100017-2] 21601096 10.1016/B978-0-12-385075-1.00017-2 PMC3121701

[60] Buckley C.E. , Moore R.E., Reade A., Goldberg A.R., Weiner O.D., Clarke J.D.W. (2016) Reversible optogenetic control of subcellular protein localization in a live vertebrate embryo. Dev. Cell, 36, 117–126. [10.1016/j.devcel.2015.12.011] 26766447 10.1016/j.devcel.2015.12.011 PMC4712025

[61] Adrian M. , Nijenhuis W., Hoogstraaten R.I., Willems J., Kapitein L.C. (2017) A phytochrome-derived photoswitch for intracellular transport. ACS Synth. Biol., 6, 1248–1256. 28340532 10.1021/acssynbio.6b00333 PMC5525101

[62] Van Audenhove I. , Van Impe K., Ruano-Gallego D., De Clercq S., De Muynck K., Vanloo B., Verstraete H., Fernández L.Á., Gettemans J. (2013) Mapping cytoskeletal protein function in cells by means of nanobodies. Cytoskeleton, 70, 604–622. 10.1002/cm.21122. 23818458 10.1002/cm.21122

[63] Beghein E. , Van Audenhove I., Zwaenepoel O., Verhelle A., De Ganck A., Gettemans J. (2016) A new survivin tracer tracks, delocalizes and captures endogenous survivin at different subcellular locations and in distinct organelles. Sci. Rep., 6, 1–16. [10.1038/srep31177] 27514728 10.1038/srep31177 PMC4981888

[64] Van Audenhove I. , Boucherie C., Pieters L., Zwaenepoel O., Vanloo B., Martens E., Verbrugge C., Hassanzadeh‐Ghassabeh G., Vandekerckhove J., Cornelissen M. et al (2014) Stratifying fascin and cortactin function in invadopodium formation using inhibitory nanobodies and targeted subcellular delocalization. FASEB J., 28, 1805–1818. 24414419 10.1096/fj.13-242537

[65] Virant D. , Traenkle B., Maier J., Kaiser P.D., Bodenhöfer M., Schmees C., Vojnovic I., Pisak-Lukáts B., Endesfelder U., Rothbauer U. (2018) A peptide tag-specific nanobody enables high-quality labeling for dSTORM imaging. Nat. Commun., 9, 1–14. 29500346 10.1038/s41467-018-03191-2 PMC5834503

[66] Zhao W. , Pferdehirt L., Segatori L. (2018) Quantitatively predictable control of cellular protein levels through proteasomal degradation. ACS Synth. Biol., 7, 540–552. 29061039 10.1021/acssynbio.7b00325

[67] Zhao W. , Bonem M., McWhite C., Silberg J.J., Segatori L. (2014) Sensitive detection of proteasomal activation using the Deg-On mammalian synthetic gene circuit. Nat. Commun., 5, 3612. 24710080 10.1038/ncomms4612

[68] Weber W. , Fux C., Daoud-El Baba M., Keller B., Weber C.C., Kramer B.P., Heinzen C., Aubel D., Bailey J.E., Fussenegger M. (2002) Macrolide-based transgene control in mammalian cells and mice. Nat. Biotechnol., 20, 901–907. 12205509 10.1038/nbt731

[69] Kramer B.P. , Viretta A.U., Baba M.D.E., Aubel D., Weber W., Fussenegger M. (2004) An engineered epigenetic transgene switch in mammalian cells. Nat. Biotechnol., 22, 867–870. 15184906 10.1038/nbt980

[70] Schindelin J. , Arganda-Carreras I., Frise E., Kaynig V., Longair M., Pietzsch T., Preibisch S., Rueden C., Saalfeld S., Schmid B. et al (2012) Fiji: an open-source platform for biological-image analysis. Nat. Methods, 9, 676–682. 22743772 10.1038/nmeth.2019 PMC3855844

[71] Caussinus E. , Kanca O., Affolter M. (2012) Fluorescent fusion protein knockout mediated by anti-GFP nanobody. Nat. Struct. Mol. Biol., 19, 117–122. 10.1038/nsmb.2180 22157958

[72] Horie C. , Suzuki H., Sakaguchi M., Mihara K. (2002) Characterization of signal that directs C-tail-anchored proteins to mammalian mitochondrial outer membrane. Mol. Biol. Cell, 13, 1615–1625. 12006657 10.1091/mbc.01-12-0570 PMC111131

[73] Anderie I. , Schulz I., Schmid A. (2007) Characterization of the C-terminal ER membrane anchor of PTP1B. Exp. Cell Res., 313, 3189–3197. 17643420 10.1016/j.yexcr.2007.05.025

[74] Brocard C. , Hartig A. (2006) Peroxisome targeting signal 1: is it really a simple tripeptide? Biochim. Biophys. Acta Mol. Cell Res., 1763, 1565–1573. 10.1016/j.bbamcr.2006.08.022 17007944

[75] Clift D. , McEwan W.A., Labzin L.I., Konieczny V., Mogessie B., James L.C., Schuh M. (2017) A method for the acute and rapid degradation of endogenous proteins. Cell, 171, 1692–1706.e18. 29153837 10.1016/j.cell.2017.10.033 PMC5733393

[76] Kalderon D. , Roberts B.L., Richardson W.D., Smith A.E. (1984) A short amino acid sequence able to specify nuclear location. Cell, 39, 499–509. 6096007 10.1016/0092-8674(84)90457-4

[77] Newman L.E. , Schiavon C., Kahn R.A. (2016) Plasmids for variable expression of proteins targeted to the mitochondrial matrix or intermembrane space. Cell. Logist., 6, 1–11. 10.1080/21592799.2016.1247939 PMC5190143 28042516

[78] Angelici B. , Mailand E., Haefliger B., Benenson Y. (2016) Synthetic biology platform for sensing and integrating endogenous transcriptional inputs in mammalian cells. Cell Rep., 16, 2525–2537. 27545896 10.1016/j.celrep.2016.07.061 PMC5009115

[79] Gossen M. , Bujard H. (1992) Tight control of gene expression in mammalian cells by tetracycline-responsive promoters. Proc. Natl. Acad. Sci. USA, 89, 5547–5551. 1319065 10.1073/pnas.89.12.5547 PMC49329

[80] Strathdee C.A. , McLeod M.R., Hall J.R. (1999) Efficient control of tetracycline-responsive gene expression from an autoregulated bi-directional expression vector. Gene, 229, 21–29. 10095100 10.1016/s0378-1119(99)00045-1

[81] Yoshida Y. , Hamada H. (1997) Adenovirus-mediated inducible gene expression through tetracycline-controllable transactivator with nuclear localization signal. Biochem. Biophys. Res. Commun., 230, 426–430. 9016796 10.1006/bbrc.1996.5975

[82] Matsuzawa S.-i. , Cuddy M., Fukushima T., Reed J.C. (2005) Method for targeting protein destruction by using a ubiquitin-independent, proteasome-mediated degradation pathway. Proc. Natl. Acad. Sci. USA, 102, 14982–14987. 16219697 10.1073/pnas.0507512102 PMC1257734

[83] Li X. , Zhao X., Fang Y., Jiang X., Duong T., Fan C., Huang C., Kain S.R. (1998) Generation of destabilized green fluorescent protein as a transcription reporter. J. Biol. Chem., 273, 34970–34975. 9857028 10.1074/jbc.273.52.34970

[84] Hillen W. , Berens C. (1994) Mechanisms underlying expression of Tn10 encoded tetracycline resistance. Annu. Rev. Microbiol., 48, 345–369. 7826010 10.1146/annurev.mi.48.100194.002021

[85] Gardner T.S. , Cantor C.R., Collins J.J. (2000) Construction of a genetic toggle switch in Escherichia coli. Nature, 403, 339–342. 10659857 10.1038/35002131

[86] Sadeghpour M. , Veliz-Cuba A., Orosz G., Josić K., Bennett M.R. (2017) Bistability and oscillations in co-repressive synthetic microbial consortia. Quant. Biol., 5, 55–66. [10.1007/s40484-017-0100-y] 28713623 10.1007/s40484-017-0100-y PMC5508549

[87] Kobayashi H. , Kaern M., Araki M., Chung K., Gardner T.S., Cantor C.R., Collins J.J. (2004) Programmable cells: interfacing natural and engineered gene networks. Proc. Natl. Acad. Sci. USA, 101, 8414–8419. 15159530 10.1073/pnas.0402940101 PMC420408

[88] Greber D. , El-Baba M.D., Fussenegger M. (2008) Intronically encoded siRNAs improve dynamic range of mammalian gene regulation systems and toggle switch. Nucleic Acids Res., 36, e101–e101. 18632760 10.1093/nar/gkn443 PMC2532736

[89] Müller K. , Engesser R., Metzger S., Schulz S., Kämpf M.M., Busacker M., Steinberg T., Tomakidi P., Ehrbar M., Nagy F. et al (2013) A red/far-red light-responsive bi-stable toggle switch to control gene expression in mammalian cells. Nucleic Acids Res, 41, 1–11. 23355611 10.1093/nar/gkt002 PMC3627562

[90] Del Vecchio D. , Sontag E.D. (2007) Dynamics and control of synthetic bio-molecular networks. In: *Proceedings of the American Control Conference*, pp. 1577–1588.

[91] Berry L.K. , Ólafsson G., Ledesma-Fernández E., Thorpe P.H. (2016) Synthetic protein interactions reveal a functional map of the cell. eLife, 5, e13053. 27098839 10.7554/eLife.13053 PMC4841780

[92] Cattaneo A. , Biocca S. (1999) The selection of intracellular antibodies. Trends Biotechnol., 17, 115–121. [10.1016/S0167-7799(98)01268-2] 10189716 10.1016/s0167-7799(98)01268-2

[93] Alessandra Vigano M. , Bieli D., Schaefer J.V., Jakob R.P., Matsuda S., Maier T., Plückthun A., Affolter M. (2018) DARPins recognize mTFP1 as novel reagents for in vitro and in vivo protein manipulations. Biol. Open, 7, 1–11. [10.1242/bio.036749] 10.1242/bio.036749 PMC6262872 30237292

[94] Decanniere K. , Desmyter A., Lauwereys M., Ghahroudi M.A., Muyldermans S., Wyns L. (1999) A single-domain antibody fragment in complex with RNase A: non-canonical loop structures and nanomolar affinity using two CDR loops. Structure, 7, 361–370. 10196124 10.1016/s0969-2126(99)80049-5

[95] Desmyter A. , Decanniere K., Muyldermans S., Wyns L. (2001) Antigen specificity and high affinity binding provided by one single loop of a camel single-domain antibody. J. Biol. Chem., 276, 26285–26290. 11342547 10.1074/jbc.M102107200

[96] Beghein E. , Gettemans J. (2017) Nanobody technology: a versatile toolkit for microscopic imaging, protein-protein interaction analysis, and protein function exploration. Front. Immunol., 8. doi:10.3389/fimmu.2017.00771. 10.3389/fimmu.2017.00771 PMC5495861 28725224

[97] Monegal A. , Ami D., Martinelli C., Huang H., Aliprandi M., Capasso P., Francavilla C., Ossolengo G., De Marco A. (2009) Immunological applications of single-domain llama recombinant antibodies isolated from a naïve library. Protein Eng. Des. Sel., 22, 273–280. 19196718 10.1093/protein/gzp002

[98] Pardon E. , Laeremans T., Triest S., Rasmussen S.G.F., Wohlkönig A., Ruf A., Muyldermans S., Hol W.G.J., Kobilka B.K., Steyaert J. (2014) A general protocol for the generation of Nanobodies for structural biology. Nat. Protoc., 9, 674–693. 24577359 10.1038/nprot.2014.039 PMC4297639

[99] Fu X. , Gao X., He S., Huang D., Zhang P., Wang X., Zhang S., Dang R., Yin S., Du E. et al (2013) Design and selection of a camelid single-chain antibody yeast two-hybrid library produced de novo for the cap protein of porcine circovirus type 2 (PCV2). PLoS One, 8, e56222. 23469171 10.1371/journal.pone.0056222 PMC3585807

[100] Schut M.H. , Pepers B.A., Klooster R., van der Maarel S.M., el Khatabi M., Verrips T., den Dunnen J.T., van Ommen G.J.B., van Roon-Mom W.M.C. (2015) Selection and characterization of llama single domain antibodies against N-terminal huntingtin. Neurol. Sci., 36, 429–434. 25294428 10.1007/s10072-014-1971-6 PMC4341019

[101] Ghahroudi M.A. , Desmyter A., Wyns L., Hamers R., Muyldermans S. (1997) Selection and identification of single domain antibody fragments from camel heavy-chain antibodies. FEBS Lett., 414, 521–526. 9323027 10.1016/s0014-5793(97)01062-4

[102] Van Der Linden R. , De Geus B., Stok W., Bos W., Van Wassenaar D., Verrips T., Frenken L. (2000) Induction of immune responses and molecular cloning of the heavy chain antibody repertoire of Lama glama. J. Immunol. Methods, 240, 185–195. [10.1016/S0022-1759(00)00188-5] 10854612 10.1016/s0022-1759(00)00188-5

[103] Sabir J.S.M. , Atef A., El-Domyati F.M., Edris S., Hajrah N., Alzohairy A.M., Bahieldin A. (2014) Construction of naïve camelids VHH repertoire in phage display-based library. C. R. Biol., 337, 244–249. 24702893 10.1016/j.crvi.2014.02.004

[104] Moutel S. , Bery N., Bernard V., Keller L., Lemesre E., De Marco A., Ligat L., Rain J.C., Favre G., Olichon A. et al (2016) NaLi-H1: a universal synthetic library of humanized nanobodies providing highly functional antibodies and intrabodies. eLife, 5, e16228. 27434673 10.7554/eLife.16228 PMC4985285

[105] Yan J. , Li G., Hu Y., Ou W., Wan Y. (2014) Construction of a synthetic phage-displayed Nanobody library with CDR3 regions randomized by trinucleotide cassettes for diagnostic applications. J. Transl. Med., 12, 1–12. 25496223 10.1186/s12967-014-0343-6 PMC4269866

[106] Tanha J. , Dubuc G., Hirama T., Narang S.A., MacKenzie C.R. (2002) Selection by phage display of llama conventional VH fragments with heavy chain antibody VHH properties. J. Immunol. Methods. doi:10.1016/S0022-1759(02)00027-3. 10.1016/s0022-1759(02)00027-3 12009207

[107] Goldman E.R. , Anderson G.P., Liu J.L., Delehanty J.B., Sherwood L.J., Osborn L.E., Cummins L.B., Hayhurst A. (2006) Facile generation of heat-stable antiviral and antitoxin single domain antibodies from a semisynthetic llama library. Anal. Chem., 78, 8245–8255. 17165813 10.1021/ac0610053 PMC2528076

[108] Dufner P. , Jermutus L., Minter R.R. (2006) Harnessing phage and ribosome display for antibody optimisation. Trends Biotechnol, 24, 523–529. [10.1016/j.tibtech.2006.09.004] 17000017 10.1016/j.tibtech.2006.09.004

[109] Wagner H.J. , Wehrle S., Weiss E., Cavallari M., Weber W. (2018) A two-step approach for the design and generation of nanobodies. Int. J. Mol. Sci., 19, 3444.10.3390/ijms19113444. 10.3390/ijms19113444 PMC6274671 30400198

[110] Van Der Linden R.H.J.J. , Frenken L.G.J.J., De Geus B., Harmsen M.M., Ruuls R.C., Stok W., De Ron L., Wilson S., Davis P., Verrips C.T. (1999) Comparison of physical chemical properties of llama VHH antibody fragments and mouse monoclonal antibodies. Biochim. Biophys. Acta Protein Struct. Mol. Enzymol., 1431, 37–46. 10.1016/s0167-4838(99)00030-8 10209277

[111] Arbabi-Ghahroudi M. , Tanha J., MacKenzie R. (2005) Prokaryotic expression of antibodies. Cancer Metastasis Rev., 24, 501–519. 16408159 10.1007/s10555-005-6193-1

[112] Peng H.-P. , Lee K.H., Jian J.-W., Yang A.-S. (2014) Origins of specificity and affinity in antibody–protein interactions. Proc. Natl. Acad. Sci. USA, 111, E2656–2665. [10.1073/pnas.1401131111] 24938786 10.1073/pnas.1401131111 PMC4084487

[113] Guilliams T. , El-Turk F., Buell A.K., O'Day E.M., Aprile F.A., Esbjörner E.K., Vendruscolo M., Cremades N., Pardon E., Wyns L. et al (2013) Nanobodies raised against monomeric α-synuclein distinguish between fibrils at different maturation stages. J. Mol. Biol., 425, 2397–2411. 23557833 10.1016/j.jmb.2013.01.040

[114] Domanska K. , Vanderhaegen S., Srinivasan V., Pardon E., Dupeux F., Marquez J.A., Giorgetti S., Stoppini M., Wyns L., Bellotti V. et al (2011) Atomic structure of a nanobody-trapped domain-swapped dimer of an amyloidogenic 2-microglobulin variant. Proc. Natl. Acad. Sci. USA, 108, 1314–1319. [10.1073/pnas.1008560108] 21220305 10.1073/pnas.1008560108 PMC3029709

[115] Rasmussen S.G.F. , Choi H.J., Fung J.J., Pardon E., Casarosa P., Chae P.S., Devree B.T., Rosenbaum D.M., Thian F.S., Kobilka T.S. et al (2011) Structure of a nanobody-stabilized active state of the β2 adrenoceptor. Nature, 469, 175–180. 21228869 10.1038/nature09648 PMC3058308

[116] Nguyen V.K. , Hamers R., Wyns L., Muyldermans S. (2000) Camel heavy-chain antibodies: diverse germline V(H)H and specific mechanisms enlarge the antigen-binding repertoire. EMBO J., 19, 921–930. 10698934 10.1093/emboj/19.5.921 PMC305632

[117] Qin J.Y. , Zhang L., Clift K.L., Hulur I., Xiang A.P., Ren B.Z., Lahn B.T. (2010) Systematic comparison of constitutive promoters and the doxycycline-inducible promoter. PLoS One, 5, e10611–6. 20485554 10.1371/journal.pone.0010611 PMC2868906

[118] Kaji H. , Canaff L., Lebrun J.J., Goltzman D., Hendy G.N. (2001) Inactivation of menin, a Smad3-interacting protein, blocks transforming growth factor type β signaling. Proc. Natl. Acad. Sci. USA, 98, 3837–3842. [10.1073/pnas.061358098] 11274402 10.1073/pnas.061358098 PMC31139

[119] Bikkavilli R.K. , Feigin M.E., Malbon C.C. (2008) p38 mitogen-activated protein kinase regulates canonical Wnt-β-catenin signaling by inactivation of GSK3β. J. Cell Sci., 121, 3598–3607. [10.1242/jcs.032854] 18946023 10.1242/jcs.032854

[120] Marasco W.A. , Haseltine W.A., Chen S. (1993) Design, intracellular expression, and activity of a human anti-human immunodeficiency virus type 1 gp120 single-chain antibody. Proc. Natl. Acad. Sci. USA, 90, 7889–7893. 8356098 10.1073/pnas.90.16.7889 PMC47248

[121] Biocca S. , Ruberti F., Tafani M., Pierandrel-Amaldi P., Cattaneo A. (1995) Redox state of single chain FV fragments targeted to the endoplasmic reticulum, cytosol and mitochondria. Bio/Technology, 13, 1110–1115. 10.1038/nbt1095-1110 9636285

[122] Moore J.D. (2013) In the wrong place at the wrong time: does cyclin mislocalization drive oncogenic transformation? Nat. Rev. Cancer, 13, 201–208. 23388618 10.1038/nrc3468

[123] Zempel H. , Mandelkow E. (2014) Lost after translation: missorting of Tau protein and consequences for Alzheimer disease. Trends Neurosci., 37, 721–732. [10.1016/j.tins.2014.08.004] 25223701 10.1016/j.tins.2014.08.004

[124] Dueber J.E. , Wu G.C., Malmirchegini G.R., Moon T.S., Petzold C.J., Ullal A.V., Prather K.L.J., Keasling J.D. (2009) Synthetic protein scaffolds provide modular control over metabolic flux. Nat. Biotechnol., 27, 753–759. [10.1038/nbt.1557] 19648908 10.1038/nbt.1557

[125] Greber D. , Fussenegger M. (2007) Mammalian synthetic biology: engineering of sophisticated gene networks. J. Biotechnol., 130, 329–345. [10.1016/j.jbiotec.2007.05.014] 17602777 10.1016/j.jbiotec.2007.05.014

