# Supplementary material for: A platform for post-translational spatiotemporal control of cellular proteins
Source: Synth Biol (Oxf). 2021 Feb 2;6(1):ysab002. doi: 10.1093/synbio/ysab002 (PMC7976946; doi:10.1093/synbio/ysab002)
Supplement: ysab002_Supplementary_Data [file ysab002_supplementary_data.zip › SI_Jayanthi_SYNBIO.pdf]

SUPPLEMENTARY INFORMATION

A platform for post-translational spatiotemporal control of cellular proteins

Brianna Jayanthi[1,†], Bhagyashree Bachhav[2,†], Zengyi Wan[3], Santiago Martinez Legaspi[2], Laura Segatori[1,2,3,4,*]

[1]Systems, Synthetic and Physical Biology Graduate Program, Rice University, Houston, Texas 77005, USA.

[2]Department of Chemical and Biomolecular Engineering, Rice University, Houston, Texas 77005, USA.

[3]Department of Bioengineering, Rice University, Houston, Texas 77005, USA.

[4]Department of Biosciences, Rice University, Houston, Texas 77005, USA.

*Corresponding author: Tel:+1 713 348 3536; E-mail: segatori@rice.edu

[†]These authors contributed equally to this work.

Supplementary Figure 1

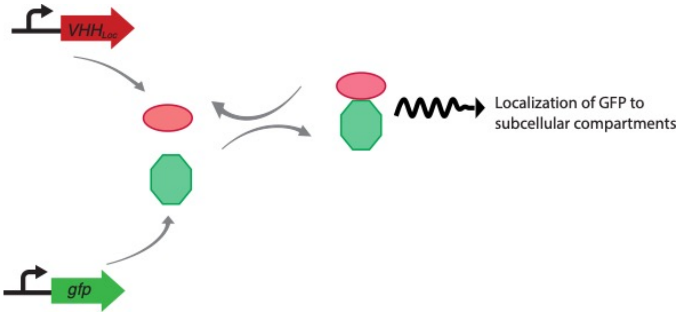

**Localization signal sequences**
1. Mitochondria outer membrane anchor (MOM)
2. Endoplasmic reticulum membrane anchor (ERM)
3. Peroxisome targeting signal (PEX)
4. Plasma membrane anchor (PM)
5. Nuclear localization signal (NLS)

Supplementary Figure 1 Schematic representation of NanoLoc-mediated control of GFP subcellular localization.

Supplementary Figure 2

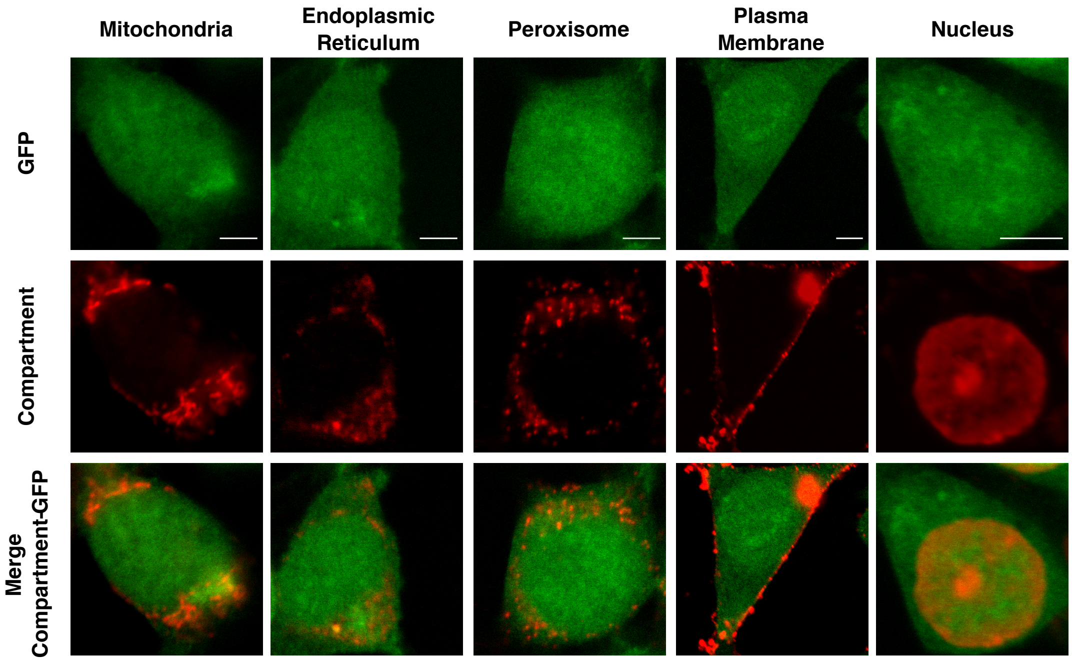

Supplementary Figure 2 GFP subcellular localization in HEK293/GFP#1 cells. Representative confocal microscopy images of HEK293/GFP#1 cells transiently transfected with a control plasmid lacking the *vhh* gene and analyzed 72 h post-transfection. GFP (green, row 1); subcellular compartment (red, row 2); colocalization of subcellular compartment and GFP shown in merged images (row 3). Scale bars: 5 μm. Brightness and contrast levels were adjusted and images of cells treated the same were subjected to the same adjustment. Pseudo-coloring was applied to the subcellular compartment stain and VHH images for the plasma membrane and the nucleus.

Supplementary Figure 3

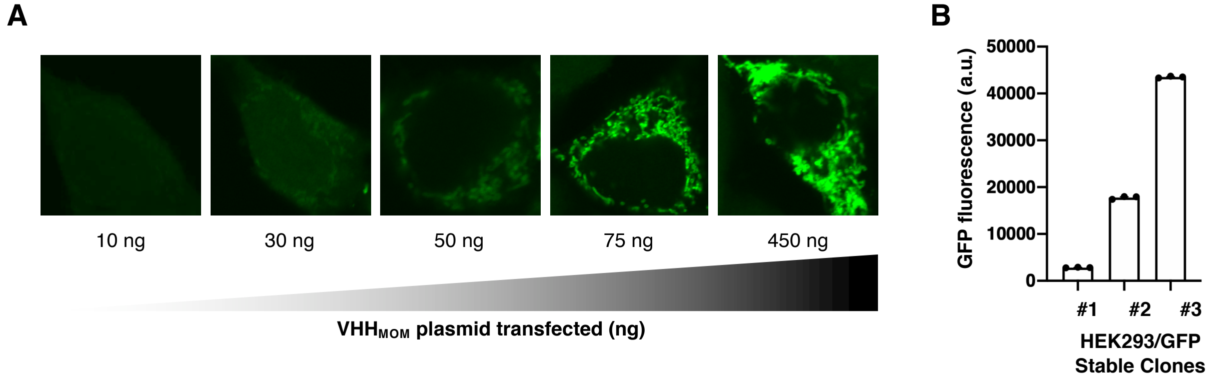

Supplementary Figure 3 GFP mitochondrial localization as a function of VHH_{MOM} expression level. (**A**) Representative images of the monoclonal HEK293 cell line (HEK293/GFP#1) transfected with a plasmid expressing VHH_{MOM} (0 - 450 ng) and analyzed 72 h post-transfection by confocal microscopy. (**B**) GFP expression level of three monoclonal stable HEK293 cell lines. Mean GFP fluorescence intensity of three HEK293/GFP stable cells measured by flow cytometry. Data are reported as mean ± s.e.m. (n = 3). Black dots represent the biological replicates.

Supplementary Figure 4

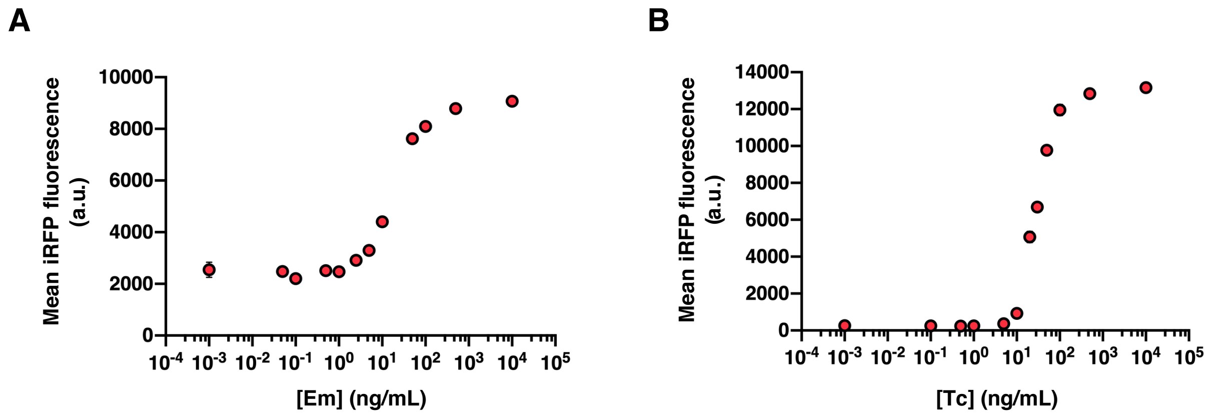

Supplementary Figure 4 Expression of iRFP from the dual-input expression system. (**A**) HEK293/GFP2R cells transiently transfected for the expression of iRFP under the control of the Em-inducible operator treated with Em (0 - 10 μg/mL) 16 h post-transfection and analyzed by flow cytometery after 48 h of Em treatment. (**B**) HEK293/GFP2R cells transiently transfected for the expression of iRFP under the control of the Tc-inducible operator treated with Tc (0 - 10 μg/mL) 16 h post-transfection and analyzed by flow cytometery after 48 h of Tc treatment. Data are reported as mean ± s.e.m. (n = 3).

Supplementary Table 1. Localization tags used in this study.

Localization tag	Amino Acid Sequence	Source
Mitochondria (MOM)	RGDGEPSGVPVAVVLLPVFALTLVAVWAFVRYRKQL	Horie et al., 2002(1)
Endoplasmic reticulum (ER)	SGLRSFLVNMCVATVLTAGAYLCYR	Anderie et al., 2007(2)
Peroxisome (PEX)	ASGYKSKL	Brocard & Hartig, 2006(3)
Plasma membrane (PM)	GCTVSAEDKAAAER	Clift et al., 2017(4)
Nuclear localization signal (NLS)	PKKKRKV	Kalderon et al., 1984(5)

Supplementary Table 2. Plasmids used in this study.

Name of the plasmid	Source/ Notes	Ref
pLenti CMV GFP Blast	Addgene	17445
Oct4-ires-EGFP (lox neo)	Addgene	21547
pLenti CMV eGFP Zeo	Addgene	17449
piRFP	Addgene	31857
pcDNA4/TO	Invitrogen	V102020
pcDNA6/TR	Invitrogen	V102520
pLenti SV40 GFP	GFP downstream of the SV40 promoter	https://benchling.com/s/seq-VEanqWt2M7smVkX0VuDC
pCMV.VHH_{MOM}	VHH tagged with mitochondrial outer membrane localization signal at the 3' end	https://benchling.com/s/seq-ssMCng8yqrss9KcvEJag
pCMV.VHH_{ERM}	VHH tagged with endoplasmic reticulum localization signal at the 3' end	https://benchling.com/s/seq-DHU05GK7ABIHpPFnD7vw
pCMV.VHH_{PEX}	VHH tagged with peroxisome localization signal at the 3' end	https://benchling.com/s/seq-0J8NErATDW8CHXtynt3r

pCMV.VHH$_{PM}$	VHH tagged with plasma membrane localization signal at the 5’ end	https://benchling.com/s/seq-jM059cRBmPfxW8ozB9lU
pCMV.VHH$_{NLS}$	VHH tagged with nuclear localization signal at the 3’ end	https://benchling.com/s/seq-2dxlwa34iHramRf5RNYU
pCMV.VHH$_{ODCwt}$	VHH tagged with ODC degron at the 3’ end	https://benchling.com/s/seq-Kq8I1iT0KW9kl236Ym3F
pCMV.VHH$_{ODC(T15A)}$	VHH tagged with ODC$_{T15A}$ degron variant at the 3’ end	https://benchling.com/s/seq-8W9aHMunXPICk9Z5neZx
pCMV.VHH$_{ODC(D12A)}$	VHH tagged with ODC$_{D12A}$ degron variant at the 3’ end	https://benchling.com/s/seq-H4qzJhcXzLQ0wfSC37G1
pCMV/ETR.iRFP	iRFP downstream of a minimal CMV promoter consisting of four repeats of the ETR operator sequence	https://benchling.com/s/seq-Zb7cBYaWWxdbFbaWeNIU
pCMV/TO.iRFP	iRFP downstream of a minimal CMV promoter consisting of two repeats of the TO operator sequence	https://benchling.com/s/seq-eAsVEfaLr7q6OBxFJGjH
pCMV/ETR.VHH$_{MOM}$	VHH-MOM downstream of a minimal CMV promoter	https://benchling.com/s/seq-ORTiwZZ7XxZgd4pPLmLX

	consisting of four repeats of the ETR operator sequence	
pCMV/TO.VHH$_{NLS}$	VHH-NLS downstream of a minimal CMV promoter consisting of two repeats of the TO operator sequence	https://benchling.com/s/seq-SKNYmQBoMbaabi9vYbmj
pCMV.TetR-IRES-EKRAB	TetR and EKRAB downstream of a CMV promoter and separated by IRES	https://benchling.com/s/seq-x4mdRMO9pvsZpNRNmLY6
pCMV/TO.VHH$_{MOM}$	VHH-MOM downstream of a minimal CMV promoter consisting of two repeats of the TO operator sequence	https://benchling.com/s/seq-AXwqMTVUfFRh74ZZtCO3
pCMV/ETR.VHH$_{ODC(wt)}$	VHH-ODC$_{wt}$ downstream of a minimal CMV promoter consisting of four repeats of the ETR operator sequence	https://benchling.com/s/seq-IGjYgJEFQxCOU0DXKUtU
pCMV/ETR.VHH$_{NLS}$-IRES-PIPKRAB	VHH-NLS and PIPKRAB separated by IRES, downstream of a CMV promoter consisting of four	https://benchling.com/s/seq-A6UVtbkshvn6lZVShzjj

	repeats of the ETR operator sequence	
pCMV/PIR.VHH$_{MOM}$-IRES-EKRAB	VHH$_{MOM}$ and EKRAB separated by IRES, downstream of a CMV promoter consisting of three repeats of the PIR operator sequence	https://benchling.com/s/seq-l0xqVhgJQhbSheKvlU5k
pTRE.GFP	plasmid encoding GFP under control of a cassette comprising 7 tetracycline operator repeats and the minimal CMV promoter	https://benchling.com/s/seq-GDeQBdEIl2hvqqlLqyX7
pCMV.tTA$_{NLS}$	tTA tagged with nuclear localization signal at the 5’ end	https://benchling.com/s/seq-xc267taLgKm3kSSFJ4tu
pCMV.TetR$_{BC2T}$	TetR tagged with BC2 peptide at the 3’ end	https://benchling.com/s/seq-GCnPzAVZCX9NQfuwcph7
pCMV.iRFP$_{BC2T}$	iRFP tagged with BC2 peptide at the 3’ end	https://benchling.com/s/seq-qEAelDSMR9nNn7CnMN5g
pCMV.tTA$_{BC2T}$	tTA tagged with BC2 peptide at the 3’ end	https://benchling.com/s/seq-kNEt50q0RuMw1KNGrdth

$\mathrm{pCMV.VHH}_{\mathrm{NLS}}^{\mathrm{BC2T}}$	$\mathrm{VHH}^{\mathrm{BC2T}}$ tagged with nuclear localization signal at the 3' end	https://benchling.com/s/seq-UtBI3edvZYfvqzEJIDnM

Supplementary Table 3. Oligonucleotides used in this study.

Primer Name	Sequence	Used For
F SV40-ClaI	ATATGTACTTATATCGATTGTGTGGAATGTGTGTCAGTTAGGG	pSV40-GFP
GFP-Rev-BamHI	AATATATATTAGGATCCCTTGTACAGCTCGTCCATGCCGAGAGTG	
BB-205R-Neo-BlpI	TTATAATATAGCTTAGCTCAGAAGAACTCGTCAAGAAGGCGA	
L_01F_pcDNA Fwd-KpnI	AAGCTTGGTACCGAGCTCGGATCCACC	
L_11F_MOM Fwd-BlpI	CCAGCGGAGCTAAGCTAGGAGGAGGCACGCGTGGGCGAGGCGACGG	pCMV-VHH-MOM
L_12R_MOM Rev-NotI	GACTCGAGCGGCCGCTTAGAGCTGCTTTCGGTATCTCACGAAGGCCCAAACTGCTACCAG	
L_09F_ERM Fwd-BlpI	CCAGCGGAGCTAAGCTAGGAGGAGGCAGTGGTCTTCGATCATTCCTGGTCAACATGTGCG	pCMV-VHH-ERM
L_10R_ERM Rev-NotI	GACTCGAGCGGCCGCTTACCTGTAGCAGAGGTAAGCGCCGGC	
L_07R_VHH Rev-PEX	GCTTACTCTTGTATCCGCTAGCGCCTCCTCCTAGCTTAGCTCCGCTGGAGACGGTGACC	pCMV-VHH-PEX
L_08R_PEX Rev-NotI	GACTCGAGCGGCCGCTTACAGCTTACTCTTGTATCCGCTAGCGCCTCCTCC	

L_15F_VHH Fwd-KpnI	CGCCACCGGTACCGGCGGAGATCAAGTCCAACTGGTGGAGTC	pCMV-PM-VHH
L_27F_VHH Fwd PM Ext	GCCGAGGACAAGGCGGCGGCCGAGCGCTTACCGGTCGCCACCGGTACCGGCGGAGATCAA	
L_25F_PM Ext-AflII	CGTTTAAACTTAAGCCACCATGGCTGCACCGTGAGCGCCGAGGACAAGGCGGCGG	
L_16R_VHH Rev-BlpI+linker-HA	TAGTCAGGCACATCGTAAGGGTAACCTCCTCCTAGCTTAGCTCCGCTGGAGACGGTGACC	
L_17R_HA ext Rev-NotI	GACTCGAGCGGCCGCTTACTACGCGTAGTCAGGCACATCGTAAGGGTAACCTCCTCC	
L_04R_VHH Rev-NES	TCCAGCTTGTTCTGCAGCTCACTGCCTCCTCCTAGCTTAGCTCCGCTGGAGACGGTGACC	pCMV-VHH-NES
L_05R_NES Ext Rev	GCTTACTTGTACGAGTCCAGATCCAACTCTTCCAGCTTGTTCTGCAGCTCACTGCCTC	
L_06R_NES Rev-NotI	GACTCGAGCGGCCGCTTACTTGTACGAGTCCAGATCCAACTCTTCCAGC	
L_02R_VHH Rev-NLS	TAGACCTTGCGCTTCTTCTTAGGGCCTCCTCCTAGCTTAGCTCCGCTGGAGACGGTGACC	pCMV-VHH-NLS
L_03R_NLS Rev-NotI	CCTCTAGACTCGAGCGGCCGCTTAGACCTTGCGCTTCTTCTTAGGGCCTCCTCC	

L_30R_TetR Rev_SbfI	CTAGAGTGCCTGCAGGTCAATAAGATCTGAATT CCCGGGATCCGCTGTACGCG	
L_31F_IRES Fwd_SbfI	GCTCCAGTCCTGCAGGCCGGGAGAGTGGGATG AATGGATATCTAACGGATCCG	
L_32R_IRES Rev_MluI	GGTAGGACACGCGTGTTGTGGCCATATTATCAT CGTGTTTTTCAAAGGAAAACCACGTCCC	pCMV.TetR_I RES_EKRAB
L_33F_EKRA B Fwd_MluI	GACCACTGACGCGTGCCACCATGCCTCGCCCA AAGTTGAAGAG	
L_34R_EKRA B Rev_SalI	GCCGTCGTGTCGACAGAGACTACCAGAGGTCG TTCCTGGCCATGC	
BL_01-HA-VHH-NLS-NheI	ATATTATAATGCTAGCCGCCACCATGTACCCTTA CGATGTGCCTGACT	
BL_02-R-NLS-AgeI	ATATTATATTACCGGTTTAGACCTTGCGCTTCTT CTTAGGG	
L_44F_IRES-Fwd_AgeI	GAACAACAACCGGTCCGGGAGAGTGGGATGAA TGGATATCTAACGG	pCMV.ETR-VHH-NLS_IRES_PIPKRAB
L_45R_IRES _Rev-AflII	GATAGACGCTTAAGGTTGTGGCCATATTATCATC GTGTTTTTCAAAGGAAAACCACG	
L_50F_PIPK RAB-Fwd_AflII	GAAATGTGCTTAAGGCCACCATGAGTCGAGGAG AGGTACGAATGGCTAAGGCAGG	

L_51R_PIPKRAB-Rev_NotI	GGTTATTGGCGGCCGCTTACCAGAGATCATTCCTTGCCATTCCTTCCATTTTAATGTCAC	
BL_05F_VHH linker Fwd_FLAG tag	GGACTACAAAGACGATGACGATAAAGGTGGTTCTTCTGGTGGTGATCAAGTCCAACTGG	pCMV.PIR-VHH-MOM_IRES_EKRAB
BL_06F_FLAG tag Fwd_KasI	GCACTAGGCGCCGCCACCATGGACTACAAAGACGATGACGATAAAGGTGGTTCTTCTGG	
BL_04R-MOM-AgeI	TTATTTATTAACCGGTTTAGAGCTGCTTTCGGTATCTCACG	
L_46F_EKRAB-Fwd_AFlII	GAATGTATCTTAAGGCCACCATGCCTCGCCCAAAGTTGAAG	
L_47R_EKRAB-Rev_NotI	GAAAAGTAGCGGCCGCCTACCAGAGGTCGTTCCTGGCCATGCC	
L_60F_L2-BC2_NotI	ATTATATAATAGCGGCCGCCTCTGGACTCAGATCTGG	pCMV-TetR-BC2T
L_61R-BC2Tag_XhoI	ATATTATATTCTCGAGTTATTGTTGCCAGTGGGAAA	
CMV Fwd	CGCAAATGGGCGGTAGGCGT	pCMV-iRFP-BC2T
L_63R_iRFP_Nostop_NotI	ATTTATATATGCGGCCGCCTCTTCCATCACGCCGATCTGC	

L_65F_tTA_NheI	ATATTATATAGCTAGCGCCACCATGTCTAGATTAGATAAAAG	pCMV-tTA-BC2T
L_64R_tTA_Nostop_NotI	ATATTATATAGCGGCCGCATAAGATCTGAATTCACCACC	
L_58F_HA-VHH(BC2T)_KpnI	ATATTAATTATGGTACCGCCACCATGTACCCTTACGATGTGC	$pCMV - VHH_{NLS}^{BC2T}$
L_59R_HA-VHH(BC2T)_BlpI	ATATTATATAGCTTAGCGCCTGAAGATGATACGGTTACCTGG	

Supplementary References

1. Horie,C., Suzuki,H., Sakaguchi,M. and Mihara,K. (2002) Characterization of signal that directs C-tail-anchored proteins to mammalian mitochondrial outer membrane. *Mol. Biol. Cell*, **13**, 1615–1625.
2. Anderie,I., Schulz,I. and Schmid,A. (2007) Characterization of the C-terminal ER membrane anchor of PTP1B. *Exp. Cell Res.*, **313**, 3189–3197.
3. Brocard,C. and Hartig,A. (2006) Peroxisome targeting signal 1: Is it really a simple tripeptide? *Biochim. Biophys. Acta - Mol. Cell Res.*, **1763**, 1565–1573.
4. Clift,D., McEwan,W.A., Labzin,L.I., Konieczny,V., Mogessie,B., James,L.C. and Schuh,M. (2017) A Method for the Acute and Rapid Degradation of Endogenous Proteins. *Cell*, **171**, 1692-1706.e18.
5. Kalderon,D., Roberts,B.L., Richardson,W.D. and Smith,A.E. (1984) A short amino acid sequence able to specify nuclear location. *Cell*, **39**, 499–509.
